# Spatiotemporal Regulation of a Single Adaptively Evolving *Trans*-Regulatory Element Contributes to Spermatogenetic Expression Divergence in *Drosophila*

**DOI:** 10.1093/molbev/msac127

**Published:** 2022-06-10

**Authors:** Yumei Huang, Rui Shang, Guang-An Lu, Weishun Zeng, Chenglong Huang, Chuangchao Zou, Tian Tang

**Affiliations:** State Key Laboratory of Biocontrol and Guangdong Key Laboratory of Plant Resources, School of Life Sciences, Sun Yat-sen University, 510275 Guangzhou, Guangdong Province, China; State Key Laboratory of Biocontrol and Guangdong Key Laboratory of Plant Resources, School of Life Sciences, Sun Yat-sen University, 510275 Guangzhou, Guangdong Province, China; State Key Laboratory of Biocontrol and Guangdong Key Laboratory of Plant Resources, School of Life Sciences, Sun Yat-sen University, 510275 Guangzhou, Guangdong Province, China; State Key Laboratory of Biocontrol and Guangdong Key Laboratory of Plant Resources, School of Life Sciences, Sun Yat-sen University, 510275 Guangzhou, Guangdong Province, China; State Key Laboratory of Biocontrol and Guangdong Key Laboratory of Plant Resources, School of Life Sciences, Sun Yat-sen University, 510275 Guangzhou, Guangdong Province, China; State Key Laboratory of Biocontrol and Guangdong Key Laboratory of Plant Resources, School of Life Sciences, Sun Yat-sen University, 510275 Guangzhou, Guangdong Province, China; State Key Laboratory of Biocontrol and Guangdong Key Laboratory of Plant Resources, School of Life Sciences, Sun Yat-sen University, 510275 Guangzhou, Guangdong Province, China

**Keywords:** *cis* and *trans* regulation, pleiotropy, selection, microRNAs, spermatogenesis

## Abstract

Due to extensive pleiotropy, *trans*-acting elements are often thought to be evolutionarily constrained. While the impact of *trans*-acting elements on gene expression evolution has been extensively studied, relatively little is understood about the contribution of a single *trans* regulator to interspecific expression and phenotypic divergence. Here, we disentangle the effects of genomic context and miR-983, an adaptively evolving young microRNA, on expression divergence between *Drosophila melanogaster* and *D. simulans*. We show miR-983 effects promote interspecific expression divergence in testis despite its antagonism with the often-predominant context effects. Single-cyst RNA-seq reveals that distinct sets of genes gain and lose miR-983 influence under disruptive or diversifying selection at different stages of spermatogenesis, potentially helping minimize antagonistic pleiotropy. At the round spermatid stage, the effects of miR-983 are weak and distributed, coincident with the transcriptome undergoing drastic expression changes. Knocking out miR-983 causes reduced sperm length with increased within-individual variation in *D. melanogaster* but not in *D. simulans,* and the *D. melanogaster* knockout also exhibits compromised sperm defense ability. Our results provide empirical evidence for the resolution of antagonistic pleiotropy and also have broad implications for the function and evolution of new *trans* regulators.

## Introduction

The evolution of gene expression and regulation is thought to underlie much of adaptation (Carroll 2000, 2008; Lemmon et al. 2014; Verta and Jones 2019; Wang et al. 2019; Diaz-Valenzuela et al. 2020; El Taher et al. 2021). Genetic variation that affects variation in gene expression occurs both in *cis* via linked polymorphisms of a gene and in *trans* through diffusible products of other genes (Wittkopp et al. 2004; Emerson and Li 2010; Signor and Nuzhdin 2018; Hill et al. 2021). Recent studies have shown that *cis* effects on gene expression are commonly predominant between species (Wittkopp et al. 2004, 2008; Shi et al. 2012; Lemmon et al. 2014), whereas *trans* effects play a larger role within species (Emerson et al. 2010; Pritchard et al. 2017; Diaz-Valenzuela et al. 2020; Josephs et al. 2020). Nevertheless, changes in *trans*-regulatory elements are shown to provide a jump-start formation of new circuits and also play a key role in the evolution of gene regulation (Lynch and Wagner 2008; Li and Johnson 2010; Jarvela and Hinman 2015; Britton et al. 2020). In contrast to considerable evidence that *cis* mutations are the primary sources of phenotypic novelty (Carroll 2000, 2008), relatively little is known about how individual *trans*-acting variants contribute to the long-term gene expression divergence and adaptation of species although the overall *trans* effects for each gene at the genomic scale are often studied for interspecies hybrids (Wittkopp et al. 2004, 2008; Shi et al. 2012; Coolon et al. 2014; Lemmon et al. 2014; Wang et al. 2019).

It is difficult to discern gene-specific *trans* effects and infer their contributions to divergence and adaptation for several reasons. First, *trans*-regulatory elements are typically highly conserved and exhibit extensive pleiotropy, which makes it difficult to compare gene-specific *trans* effects on phenotypes between species. Compared with *cis*-regulatory elements, *trans*-regulatory elements are more often subject to antagonistic pleiotropy, in which a gene has opposing effects on different components of fitness (Wagner and Zhang 2011; Qian et al. 2012; Chen and Zhang 2020). Yet antagonistic pleiotropy is known to limit the extent and rate of adaptation (Fisher 1930; Orr 2000). Second, empirical evidence shows that compensation between *cis* and *trans* effects, when they occur simultaneously and affect target gene expression in opposite directions, is prevalent and likely resulted from stabilizing selection (Shi et al. 2012; Signor and Nuzhdin 2018; Bao et al. 2019). So, it is difficult to infer the contribution of *trans* effects to adaption at the expression level. Third, the common approaches used to disentangle *cis*- and *trans*-effects provide little insight into the specific genetic changes underlying regulatory variation (Hill et al. 2021). While the allele-specific gene expression approach using F1 hybrids can assign the effects of all *cis* or *trans* elements to each target gene, it cannot disentangle the effect of each single regulatory element. By contrast, the expression quantitative trait loci (eQTL) mapping approach using panels of genetically different individuals shuffled by recombination can capture the regulatory effect of individual loci but eQTLs often span relatively large genomic regions, making identifying causal variants difficult (Hill et al. 2021).

Here, we study the relative contributions of a single *trans*-regulatory element and the genomic context to testis gene-expression divergence between *D. melanogaster* and *D. simulans.* Specifically, we addressed how *trans*-regulatory elements respond to positive selection avoiding antagonistic pleiotropy. To overcome the caveats of previous approaches, we used gene replacement and single-cyst RNA-seq analysis. The *trans*-regulatory element we analyzed is an adaptively evolving microRNA (miRNA), miR-983, which shows sequence divergence between *Drosophila* species (Lyu et al. 2014; Mohammed et al. 2014), possesses a large number of targets, exhibits testis-preferential expression (Lyu et al. 2014; Mohammed et al. 2014; Mohammed et al. 2018), and affects different components of male fitness (Lu et al. 2018; Zhao et al. 2021) so is suitable for this study. Our results suggest that despite antagonism with the often-predominant *cis* effects, *trans* regulators may reduce pleiotropy and respond to natural selection via spatiotemporally specific regulation. Such regulation is likely determined by the dynamic expression of genes affected, rather than the regulators.

## Results

### MiR-983 Exerts Divergent Regulation between *Drosophila* Species

**Fig. 1.**
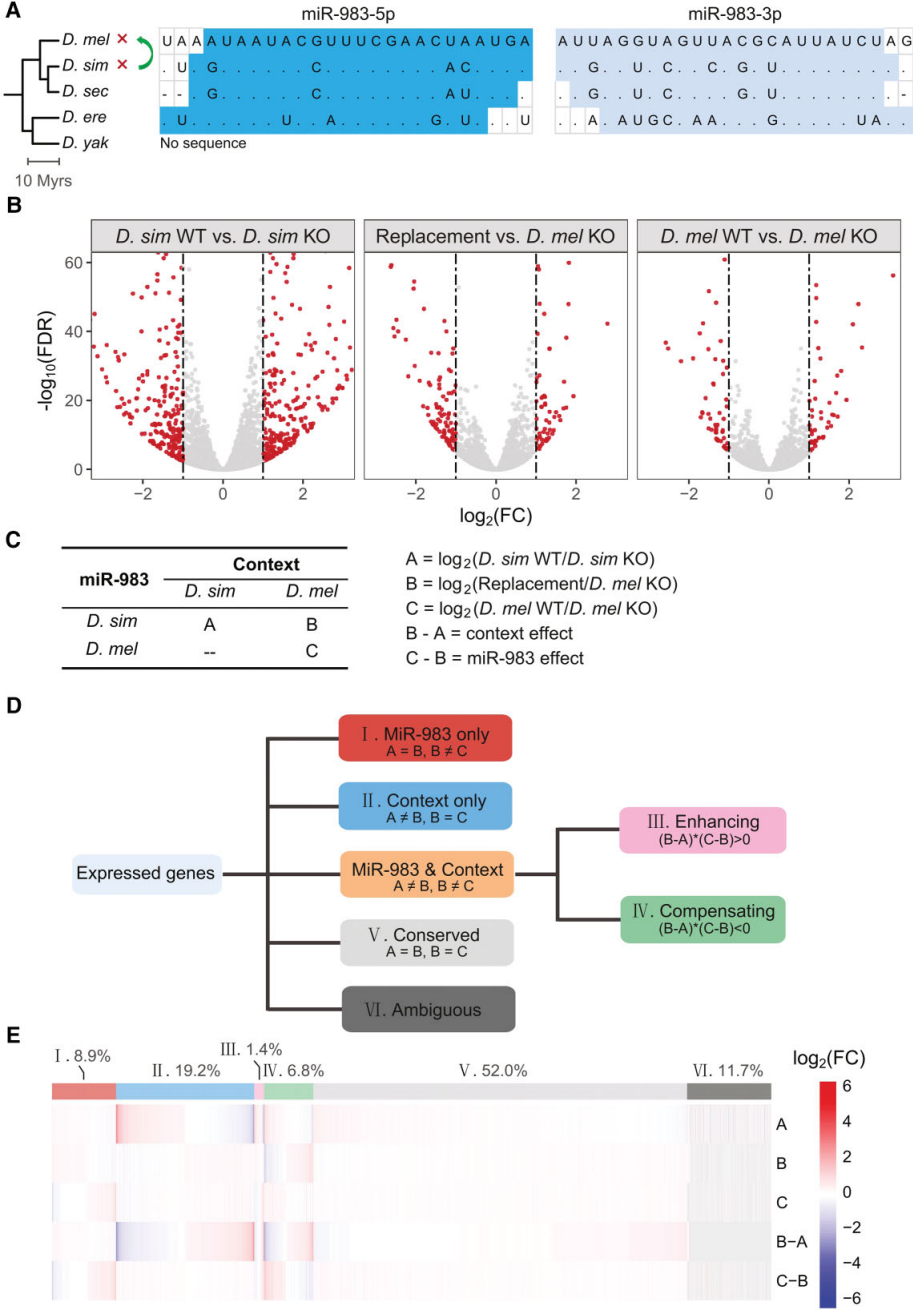
Disentangling the regulatory effects of miR-983 and the genomic context on testis gene expression. (*A*) Alignment of the mature sequences of miR-983. 5p and 3p arms are indicated separately. The phylogenetic tree of five *Drosophila* sibling species adopted from TimeTree (Kumar et al. 2017), constituting *melanogaster* subgroup, is shown on the left and scaled in million years (Myrs). Symbols to the right of the tree indicate the genetic manipulations of miR-983 used in this study: cross, knocking out; curved arrow symbol, gene replacement. Species abbreviations: *D. mel*, *D. melanogaster*; *D. sim*, *D. simulans*; *D. sec*, *D. sechellia*; *D. ere*, *D. erecta*; *D. yak*, *D. yakuba*. (*B*) Volcano plot showing the numbers and magnitudes of differentially expressed genes with |log_2_FC| > 1 and FDR < 0.05. The dotted lines denote 2-fold difference. (*C*) Explanation of each regulatory effect. (*D*) Gene classification flow chart based on regulatory effects shown in (*C*). (*E*) Clustered heatmap showing the magnitude of regulatory effects on each expressed gene. The proportions of genes in each category are denoted above.

MiR-983 is an X-linked and testis-preferential new miRNA that is found only in the *melanogaster* subgroup (Lyu et al. 2014; Mohammed et al. 2014; Mohammed et al. 2018) (fig. 1*A*). We and others previously reported that miR-983 has evolved adaptively in both *D. melanogaster* and *D. simulans* (Lyu et al. 2014; Mohammed et al. 2014). Consistent with the expectation of distinct seed sequences, which are crucial for target recognition (Agarwal et al. 2018), loss-of-function mutants of miR-983 exhibit divergent mis-expression of genes between *D. melanogaster* and *D. simulans* (Zhao et al. 2021). However, it is unclear whether such divergence is caused by changes in miR-983 or in genomic contexts. To disentangle the effects of miR-983 and genomic contexts, we re-knocked out *mir-983* in *D. melanogaster* (*dme-mir-983-1/-2*) and replaced it with the orthologous copy from *D. simulans* (*dsi-mir-983a*) using a CRISPR-Cas9 system (fig. 1*A*; supplementary fig. S1A, Supplementary Material online; also see Supplementary Methods), resulting in three *D. melanogaster* strains of miR-983 knockout (KO), replacement, and wild type (WT). The previously generated *dsi-mir-983a* KO line in *D. simulans* and its control (Zhao et al. 2021) were also included in this study (fig. 1*A*; supplementary fig. S1A, Supplementary Material online). The complete removal of miR-983 in both species was confirmed by Sanger sequencing and quantitative real-time polymerase chain reaction (qRT-PCR) (supplementary fig. S1B, Supplementary Material online).

Rather than a few predefined traits chosen to infer antagonistic pleiotropy, gene expression can be powerful molecular traits for addressing how miR-983 responds to natural selection despite antagonistic pleiotropy. While stabilizing selection would reduce divergence of gene expression between species, resulting in compensating *cis* and *trans* effects acting in opposite directions (Shi et al. 2012; Signor and Nuzhdin 2018; Bao et al. 2019), disruptive or diversifying selection would promote expression divergence, leading to enhancing *cis* and *trans* effects acting in the same direction (Shi et al. 2012; Signor and Nuzhdin 2018; Bao et al. 2019). To disentangle miR-983 effects (considered as *trans*-acting) and genomic context effects (considered as *cis*-acting) on expression divergence, we sequenced 15 adult testis RNA-seq libraries including three biological replicates each from the five strains used in this study (supplementary table S1, Supplementary Material online). Levels of gene expression were measured as normalized mean counts by DESeq2 (Love et al. 2014). Genes with more than 5 normalized mean counts on average among replicates in at least one genotype were considered expressed. Among the 12,378 genes that are 1:1 orthologous between *D. melanogaster* and *D. simulans* (http://flybase.org/), 11,299 were expressed and retained for further analyses while the rest were discarded. Consistent with the previous report that the null allele of miR-983 affects testis transcriptome more in *D. simulans* than in *D. melanogaster* (Zhao et al. 2021), more genes were differentially expressed (|log_2_FC| > 1 and FDR < 0.05; FC, fold change; FDR, false discovery rate) between miR-983 KO and control in *D. simulans* (559) than in *D. melanogaster* (115, fig. 1*B*). The magnitude of mis-expression in the replacement line is somewhere in between (172) (fig. 1*B*). These results suggest that miR-983 exerts different regulation in *D. simulans* and *D. melanogaster*.

### Disentangling MiR-983 and Genetic Context Effects on Gene Expression

The observed expression changes between control and KO lines in both species reflect a combination of miR-983 and genomic background effects. By contrast, the replacement line isolates the *D. simulans* miR-983 action, while keeping the *D. melanogaster* genomic context. To decompose miR-983 effects and context effects on testis gene expression, we compared log_2_ ratio of WT/KO normalized mean counts in *D. simulans* (A = log_2_(*D. sim* WT/*D. sim* KO)) and *D. melanogaster* (C = log_2_(*D. mel* WT/*D. mel* KO)) with log_2_ ratio of replacement/*D. mel* KO normalized mean counts (B = log_2_(replacement/*D. mel* KO)) (fig. 1*C*; see Materials and Methods). Each of the three miR-983-containing genotypes was compared to a KO strain with matching genetic background. Context effects (B-A), including the *cis* effect and the context *trans* effect except for miR-983, were measured as the difference in expression change between lines with or without dsi-miR-983 in different genetic backgrounds (B for *D. melanogaster* and A for *D. simulans*, fig. 1*C*). The log2 fold changes of A, B, and C as calculated by DEseq2 were compared by a simple subtraction followed by a two-tailed Student's *t*-test. Significant expression differences between A and B (A ≠ B; two-tailed Student's *t*-test, *P* < 0.05) would reflect context effects (fig. 1*C*). By contrast, direct miR-983 effects (C-B) were measured as the difference in expression change between lines with the presence of different miR-983 orthologs (C for dme-miR-983 and B for dsi-miR-983) relative to KO in the *D. melanogaster* background (fig. 1*C*). Significant expression differences between B and C (B ≠ C; two-tailed Student's *t*-test, *P* < 0.05) would reflect miR-983 effects (fig. 1*C*).

Based on these criteria (fig. 1*C*), expressed genes were assigned into one of six categories (fig. 1*D*; supplementary fig. S2A, Supplementary Material online). About 11.7% of the total genes (1,322 of 11,299) are uninformative (category VI) and were thus removed from further analysis unless stated otherwise. In addition, 1,006 (∼8.9%) genes were associated with only miR-983 effects (A = B and B ≠ C, category I), which is about half of the genes affected only by the genetic background (2,169 or ∼19.2%, A ≠ B and B = C, category II; fig. 1*E*). An additional 930 (∼8.2%) genes were affected by both miR-983 and genomic context (fig. 1*E*). When miR-983 and context effects were both detected, genes were further divided according to whether the two effects acted in the same direction (enhancing) or in opposite directions (compensating) in governing gene expression (fig. 1*D*). There are four times more genes under compensating (category IV) than enhancing (category III) miR-983 and context effects (fig. 1*E*), consistent with the notion that stabilizing selection is the primary evolutionary force governing the evolution of gene expression (Denver et al. 2005; Rifkin et al. 2005; Gilad et al. 2006; Bedford and Hartl 2009; Coolon et al. 2014; Huang et al. 2016; Signor and Nuzhdin 2018). The remaining 5,872 genes (∼52.0%) not affected by either miR-983 or genomic context (A = B and B = C; category V, fig. 1*E*) were considered to be under conserved regulation (fig. 1*D*).

### MiR-983 Contributes to Testis Expression Divergence Despite Antagonism with Context Effects

**Fig. 2.**
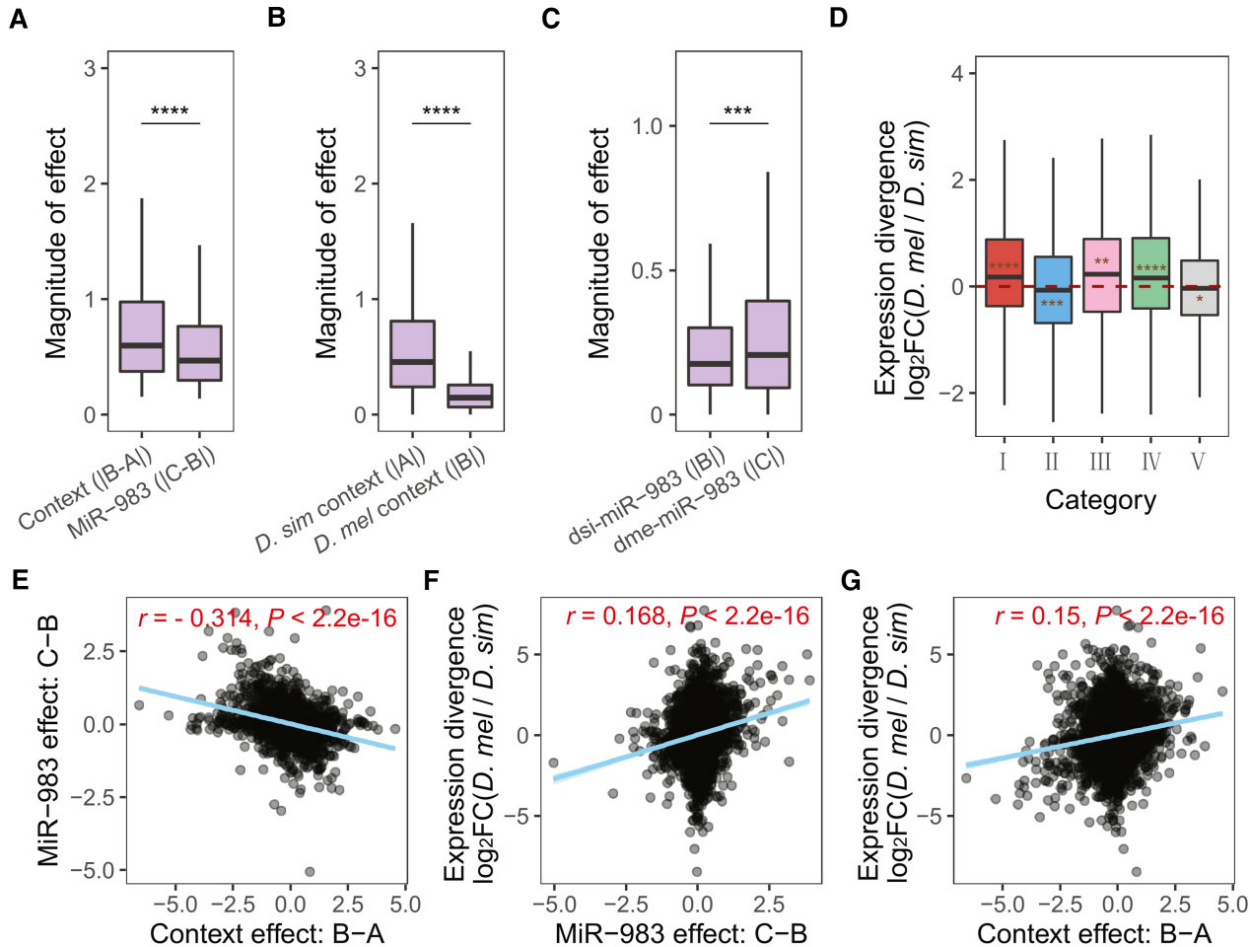
Regulatory effects of miR-983 and genomic context on gene expression in testis. (*A–C*) Comparison of regulation magnitudes. (*A*) Context effects compared to miR-983 regulation, based on genes under the influence of both the genomic context and miR-983. (*B*) The regulatory magnitudes of dsi-miR-983 in *D. simulans* compared to *Drosophila melanogaster* using genes with only context effects. (*C*) The regulatory magnitudes of dme-miR-983 in *D. melanogaster* compared to dsi-miR-983 focusing on genes with only miR-983 effects. Wilcoxon rank-sum tests were performed for statistical comparisons for (*A–C*) (***: *P* < 0.001, ****: *P* < 0.0001). (*D*) Interspecific expression divergence of genes across regulatory categories. Significant deviations from zero, as indicated by asterisks, were inferred by two-tailed Student's *t*-tests (*: *P* < 0.05, **: *P* < 0.01, ***: *P* < 0.001, ****: *P* < 0.0001). Asterisks above or below the horizontal line *y* = 0 indicate the mean is larger or less than zero. (*E-G*) Correlation between regulatory components. (*E*) miR-983 effect versus context effect. (*F*) Interspecific expression divergence versus miR-983 effect. (*G*) Interspecific expression divergence versus context effect. Pearson's correlation coefficients *r* and *P* values are shown.

We now sought to assess the relative contributions of miR-983 effects and context effects to testis expression divergence between *D. melanogaster* and *D. simulans*. To this end, we first compared the magnitudes of miR-983 effects and context effects. In line with the previous observation that *cis* effects are predominant between species (Wittkopp et al. 2004, 2008; Shi et al. 2012; Lemmon et al. 2014), we found significantly stronger genomic context effects than miR-983 effects (|B - A| > |C - B|, Wilcoxon rank-sum test, *P* = 1.9 × 10^−12^) among genes under both miR-983 and background control (categories III and IV; fig. 2*A*). Stronger effects of context than miR-983 were also observed when considering genes regulated solely by genomic context (category II) and those by miR-983 only (category I; supplementary fig. S2*B*, Supplementary Material online). Nevertheless, expression changes between the WT or replacement and miR-983 KO were significantly greater in the conspecific than the interspecific context for both genes with only context effects (fig. 2*B*) and genes with only miR-983 effects (fig. 2*C*). We observe in parallel that dsi-miR-983 effects are stronger in *D. simulans* than in *D. melanogaster* (Wilcoxon rank-sum test, *P* < 2.22 × 10^−16^; fig. 2*B*) and dme-miR-983 exerts stronger effects than dsi-miR-983 within *D. melanogaster* (Wilcoxon rank-sum test, *P* < 0.001; fig. 2*C*), suggesting that miR-983 has coevolved with the transcriptomic context within each *Drosophila* species.

We then measured expression divergence for each gene as the relative expression level (log_2_FC) between WTs of *D. melanogaster* and *D. simulans*. Genes affected by miR-983 effects (categories I, III and IV) were expressed higher in *D. melanogaster* than *D. simulans* on average (two-tailed Student's *t*-test, all *P* < 0.01, fig. 2*D*). In contrast, genes regulated by genomic context effects only (category II) or with conserved regulation (category V) tend to be up-regulated in *D. simulans* compared to *D. melanogaster* (two-tailed Student's *t*-test, both *P* < 0.05, fig. 2*D*), suggesting an antagonistic relationship between miR-983 effects and genomic context effects. We did not observe elevated magnitude of expression divergence for any gene category involving miR-983 effects, albeit genes with conserved regulation exhibited significantly lower expression divergence than the others (supplementary fig. S3, Supplementary Material online). Accordingly, there is a modest negative correlation between miR-983 and context effects on each gene (Pearson's *r* = −0.314, *P* < 2.2 × 10^−16^; fig. 2*E*; see also targets in supplementary fig. S2C, Supplementary Material online), consistent with previous reports of the prevalent antagonism between *cis* and *trans* effects (Shi et al. 2012; Signor and Nuzhdin 2018; Bao et al. 2019). When performing the cross-replicate approach to eliminate potential computational artifacts (Fraser 2019), the negative miR-983-context correlations become even stronger for both the whole transcriptome and predicted targets (supplementary table S2, Supplementary Material online).

Both miR-983 and context effects positively correlated with expression divergence between *D. simulans* and *D. melanogaster.* The extent of correlation is slightly higher for miR-983 effects (Pearson's correlation, *r* = 0.168, *P* < 2.2 × 10^−16^, fig. 2*F*) than context effects (Pearson's correlation, *r* = 0.150, *P* < 2.2 × 10^−16^; fig. 2*G*), suggesting miR-983, as expected, is distinguished from the genomic background in its contribution to expression divergence. Moreover, predicted target genes (stronger target set; see Materials and Methods) of miR-983 exhibit stronger positive correlation of expression divergence with the magnitude of miR-983 regulation (Pearson's *r* = 0.279, *P* < 2.19 × 10^−10^; supplementary fig. S2D, Supplementary Material online) than with the strength of context effects (Pearson's *r* = 0.101, *P* = 0.0244; supplementary fig. S2E, Supplementary Material online), suggesting that miR-983 effects exert directly on target genes. Taken together, miR-983 does make a significant contribution to testis expression divergence between *D. melanogaster* and *D. simulans* despite its antagonism with the often-predominant context effects. Although genes with enhancing miR-983 and context effects are expected to have elevated expression divergence due to disruptive or diversifying selection, such a pattern cannot be verified using the whole testis data.

### Transient Usage of MiR-983 Regulation across Stages of Spermatogenesis

**Fig. 3.**
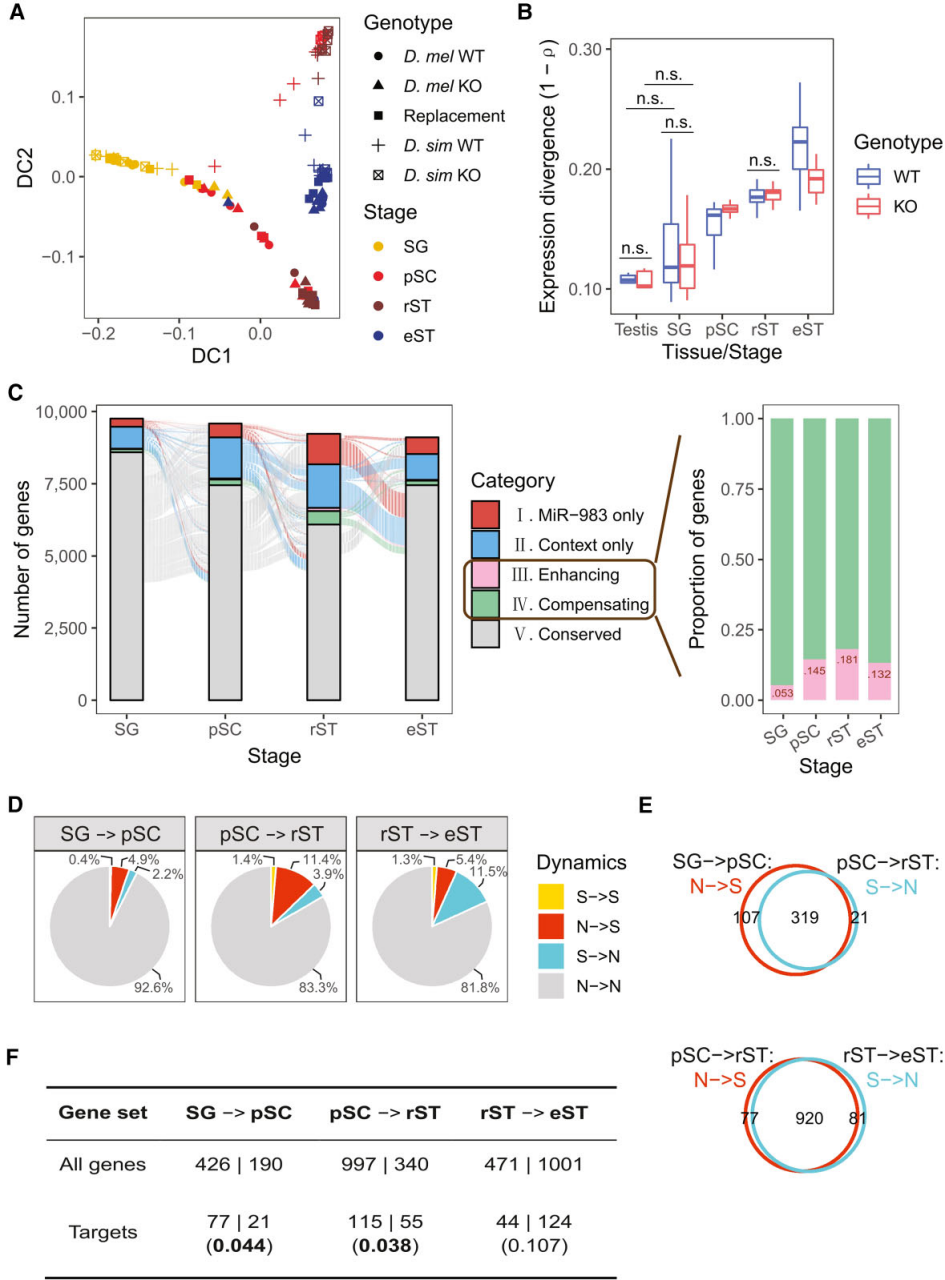
Regulatory dynamics of miR-983 across spermatogenesis. (*A*) Diffusion map for expression abundance of genes in single-cyst samples. (*B*) Global interspecific expression distance between *Drosophila melanogaster* and *D. simulans,* calculated as 1 − *ρ* (Spearman's correlation coefficient), for WT or KO strains. All pairwise comparisons among tissue/stages or between WT and KO in the same tissue/stage are significant (Wilcoxon rank-sum test: *P* < 0.01) unless indicated (by n.s.) in the plots. (*C*) Alluvial plot on the left depicting regulatory mode changes during spermatogenesis of each gene with unambiguous miR-983-mediated regulation. Bar plot on the right showing the proportion distributions of genes with compensating or enhancing miR-983 and context interactions. (*D*) Pie charts showing proportions of four types of regulatory dynamics between successive stages. “S” denotes “miR-983 only” and “enhancing” categories, while “N” represents “context only,” “compensating,” and “conserved.” (*E*) Venn plots showing the turnover of regulatory mode between successive periods. (*F*) The numbers of genes gaining (N **→** S) versus losing “S” (S **→** N) between successive spermatogenic stages. Ratios are compared between targets and all genes (those with unambiguous miR-983-mediated regulation at all the four spermatogenic stages). χ^2^ tests were performed, and *P* values are shown in parentheses.

Response to selection would be enhanced if miR-983 acts in cell-type or stage-specific manner during spermatogenesis, avoiding extensive pleiotropy. To test this hypothesis, we sequenced 107 SMART-seq2 libraries from single spermatogenic cysts (fig. 3*A*) from each of the five lines, representing four stages of spermatogonium (SG), late primary spermatocyte (pSC), round spermatid (rST), and elongating spermatid (eST), each with four to six biological replicates (supplementary fig. S4*A*, Supplementary Material online; supplementary table S1, Supplementary Material online). Spermatogenesis is a dynamic and highly organized developmental process that occurs in the testis (Fuller 1998; White-Cooper 2004). Sperms are derived from diploid spermatogonial stem cells, which differentiate to generate spermatogonia (SG). The differentiated SG further develops into pSC before entering meiosis. Through meiosis, haploid rSTs are generated with dramatic changes in morphology and physiology (Fuller 1998; White-Cooper 2004). rST further develops into eST and finally spermatozoa with extreme chromatin condensation. Staging of these spermatogenic cysts was confirmed by checking expression patterns of previously reported stage-specific *Drosophila* spermatogenesis marker genes (supplementary fig. S4*B*, Supplementary Material online). The numbers of 1 : 1 orthologous genes expressed in at least one genotype at individual stages (11,376 at the SG stage, 11,247 at pSC, 11,030 at rST, and 10,953 at eST) are comparable to the number of genes detected by RNA-seq of whole adult testes, indicating the high quality of our single-cyst RNA-seq data. Accordingly, diffusion map analysis grouped the cyst samples in a sequential order of spermatogenetic stages by species but not genotypes (fig. 3*A*). *D. melanogaster* and *D. simulans* show different developmental trajectories since the pSC stage (fig. 3*A*).

Expression divergence between the wildtype *D. melanogaster* and *D. simulans*, measured as 1 − Spearman's correlation coefficient (*ρ*), increased gradually during spermatogenesis (fig. 3*B*), and it was greater than that of the whole testis at all stages except for the SG stage (Wilcoxon rank-sum test, *P* < 0.01; fig. 3*B*). Interspecific expression divergence of miR-983 KO exhibited the same trend as that of the WT (fig. 3*B*), suggesting the increase of expression divergence during spermatogenesis is mainly attributable to programmed changes of spermatogenetic transcriptomes. Yet, the difference of expression divergence between the WT and miR-983 KO also varied across the four spermatogenetic stages (fig. 3*B*), indicating a dynamic role of miR-983 in modulating expression divergence during spermatogenesis.

To assess the dynamic effects of miR-983 on expression divergence, we disentangled miR-983 and context effects at each spermatogenetic stage using the same method as described above (fig. 3*C*). Nine-thousand one-hundred twelve to 9,755 expressed genes can be unambiguously classified into categories based on miR-983 and/or context effects, with 8,730 genes common among all stages. Consistent with the expectation that less differentiated cells exhibit more conserved expression, the proportion of genes with conserved regulation (category V) is the largest at the SG stage (fig. 3*C*). In contrast, such proportion is the lowest at the rST stage (fig. 3*C*), accordant with the fact that rST cells are generated with dramatic changes through meiosis. Cysts at the rST stage also exhibited a sharp increase in the proportion of genes affected only by miR-983 (category I, 9.7%; fig. 3*C*) and the highest proportion of genes with enhancing miR-983 and context effects (0.93%, category III; fig. 3*C*). Intriguingly, genes with enhancing miR-983 and context effects (category III) exhibited globally higher level of expression divergence (|log_2_FC|) between the wildtype *D. melanogaster* and *D. simulans* than other genes at all stages (supplementary fig. S3, Supplementary Material online), particularly for the SG, suggesting disruptive or diversifying selection indeed promotes expression divergence as we expected.

The dynamics of miR-983 regulation under selection can be revealed by changes in regulatory modes during spermatogenesis. We focused on genes with only miR-983 effects (category I) and enhancing miR-983 and context effects (category III) because expression of these genes was likely subject to the same selective pressure for adaptive miR-983 sequence evolution. We denoted these two categories of genes as “S” for under selection and the remaining genes were denoted as “N”. Genes with changing regulatory mode classification from N to S were most abundant between pSC and rST (997 of 8,730 genes, 11.4%; fig. 3*D*) while regulatory mode change from S to N was most prominent between rST and eST (1001 of 8,730 genes, 11.5%; fig. 3*D*). Interestingly, a large fraction of genes that gained the S regulatory mode at the pSC (74.9%) and rST stage (92.3%) lost it at the next stage (Hypergeometric test, both *P* = 0; fig. 3*E*). Only 10 genes maintained the S mode consistently during spermatogenesis (supplementary table S3, Supplementary Material online) whereas most genes (6,680 of 8,730 genes, 76.5%) maintained the N mode (fig. 3*C*). A similar regulatory mode change pattern was observed among predicted miR-983 targets (supplementary fig. S5, Supplementary Material online). Predicted miR-983 targets tend to gain the S mode at pSC and lose it at the rST stage (χ^2^ test, both *P* < 0.05, fig. 3*F*). These results suggest that miR-983 regulation under selection is highly dynamic and often stage-specific for different gene sets as the cells progress from primary spermatocytes to spermatids.

Gene ontology analysis revealed that genes that transiently gained the S mode at the pSC stage (319) are enriched in functional categories involved in biogenesis and cellular organization crucial for spermatocyte meiosis, while genes that transiently attained the S mode at the rST stage (920) are enriched in functional categories involved in spermatid morphogenesis and energy metabolism (supplementary fig. S6, Supplementary Material online). In addition, the 471 genes that gained the S mode at the eST stage are enriched in functional categories associated with cilium movement (supplementary fig. S6, Supplementary Material online). These results suggest that the dynamic regulation by miR-983 is pertinent to the key biological processes at individual stages. By contrast, the 10 genes maintaining S mode throughout the spermatogenesis are enriched in regulation of essential biological processes such as nitrogen compound metabolism and transcription (supplementary fig. S6, Supplementary Material online and supplementary table S3, Supplementary Material online). Eight of the ten genes are only affected by miR-983 effects at all four stages but only two were predicted targets of miR-983, suggesting the vast majority of genes affected might be subject to secondary effects in the cascade of miR-983 regulation rather than direct targets.

### Regulatory Mode Changes of MiR-983 Underlie Expression Divergence during Spermatogenesis

**Fig. 4.**
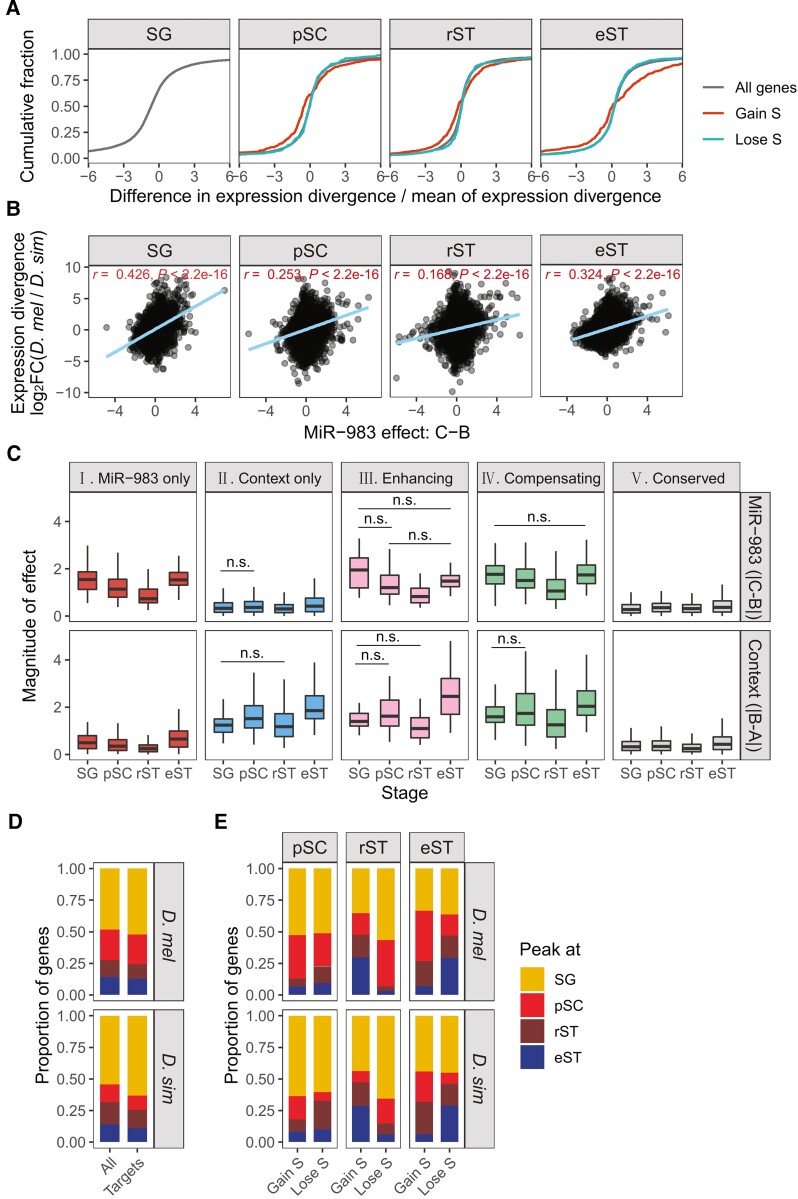
The dynamics of miR-983 regulation and transcriptome context during spermatogenesis. (*A*) Cumulative distribution plots showing the distribution of difference in interspecific expression divergence between miR-983 KO and WT strains normalized by their mean values. The interspecific expression divergence was calculated as log_2_ fold changes of gene expression in *Drosophila melanogaster* versus that in *D. simulans* for miR-983 KO and WT strains separately. “S” denotes gene categories “miR-983 only” (I) and “enhancing” (III) as in fig. 3. (*B*) Correlation between interspecific expression divergence and miR-983 regulatory effect. Pearson's correlation coefficients *r* and *P* values are shown. (*C*) Strength of miR-983 effect (first row) or context effect (second row). All pairwise comparisons among stages in the same panel are significant (Wilcoxon rank-sum test: *P* < 0.05) unless indicated (by n.s.) in the plots. (*D*) Gene proportion distributions of expression trends, which culminate at the stages of SG, pSC, rST, or eST. The union set of expressed genes at all four stages are denoted as all genes. The same colors are used in (*D,E*). (*E*) Proportion distributions of genes gaining or losing the S regulatory mode at corresponding stages. The “S” mode represents “miR-983 only” and “enhancing” regulatory categories.

If selection favors stage-specific miR-983 regulation for the increase of expression divergence, we would expect that genes gaining S mode (N **→** S) at each stage exhibit larger expression divergence than the transcriptome on average. We measured the overall contribution of miR-983 to gene expression divergence (log_2_FC) between *D. melanogaster* and *D. simulans* for individual genes as the difference of interspecific expression divergence between miR-983 KO and the WT normalized by the mean of expression divergence of miR-983 KO and the WT (fig. 4*A*). The more miR-983 contributes to expression divergence, the greater difference of expression divergence between miR-983 KO and the WT. As expected, genes gaining S mode possess a larger proportion of loci with high expression-divergence difference than the whole transcriptome and genes that lost it (N **→** S) at all of the pSC, rST, and eST stages (Kolmogorov-Smirnov test, all *P* < 0.001, fig. 4*A*), suggesting that gains of miR-983 regulation increase expression divergence during spermatogenesis.

We then compared miR-983 effects on expression divergence between stages. At the SG stage when genes with conserved regulation are most abundant (fig. 3*C*), miR-983 effects are strongly correlated with the interspecific expression divergence of WTs (Pearson correlation *r* = 0.426, *P* < 2.2 × 10^−16^; fig. 4*B*) and most prominent for genes with enhancing miR-983 and context effects (supplementary fig. S3, Supplementary Material online). In contrast, at the rST stage when genes gaining S mode are the most abundant (fig. 3*D*), miR-983 effects exhibit a weaker correlation with interspecific expression divergence of WTs (Pearson correlation, *r* = 0.168, *P* < 2.2 × 10^−16^, fig. 4*B*) compared with other stages (fig. 4*B*). In fact, the magnitudes of both miR-983 effects (|C-B|) and context effects (|B-A|) are globally lower for all gene categories at rST compared with other stages (fig. 4*C*), suggesting that the unexpected weak miR-983 effects at the rST stage is determined by the characteristics of transcriptome context. rSTs are formed at the completion of meiosis. These spermatids then undergo dramatic morphological and physiological changes and thus are expected to experience drastic transcriptomic changes as well.

To characterize the expression changes pertinent to miR-983 regulation, we compared changes in expression patterns during spermatogenesis between genes that gained and lost S mode at each stage. Using the unsupervised soft clustering method from the package Mfuzz (Kumar and Futschik 2007), the gene expression trajectories were clustered into one of the four clusters in which peak expression of the expressed genes is found at the SG (cluster I), pSC (cluster II), rST (cluster III), or eST stage (cluster IV). Consistent with the known expression pattern of spermatogenesis (Witt et al. 2019; Mahadevaraju et al. 2021), most genes reached peak expression at the SG stage and expression levels then declined (cluster I, fig. 4*D*). Genes that gained the S mode at the rST stage are more likely than those losing S mode to reach peak expression at the meiotic or post-meiotic stages (clusters II-IV) in both *D. melanogaster* (χ^2^ test, *P* = 4.461 × 10^−11^) and *D. simulans* (χ^2^ test, *P* = 2.52 × 10^−11^, fig. 4*E*). However, no such difference was found between genes gaining and losing S mode at the pSC or eST stage (χ^2^ test, all *P* > 0.05, fig. 4*E*). These results suggest that for genes affected by miR-983, expression changes are more drastic at rST than other stages. Taken together, the weak and distributed effects of miR-983 at the rST stage are coincident with extensive expression changes of the genes it affected.

### Phenotypic Effects of MiR-983 on Male Reproduction and Potential Genes Involved

**Fig. 5.**
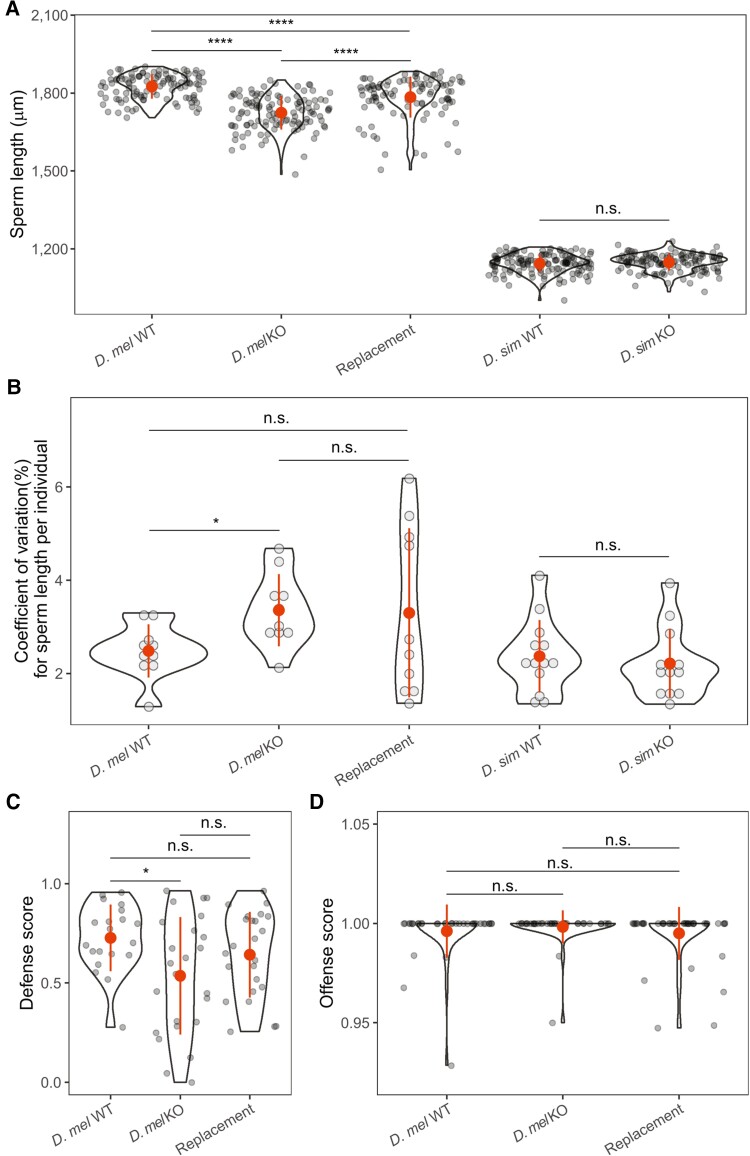
Sperm length and relative sperm competitive ability of miR-983 mutant strains. (*A*) Sperm length of WT and miR-983 mutants of *Drosophila melanogaster* and *D. simulans*. Each data point represents one sperm. *N_D. mel_*_WT_ = 109, *N_D. mel_*._KO_ = 116, *N*_Replacement_ = 100, *N_D. sim_*_WT_ = 142, *N_D. sim_*_KO_ = 132. (*B*) CV of sperm length within individual for WT and miR-983 mutants of *D. melanogaster* and *D. simulans*. Each data point represents a male individual and 8-12 sperms per male were examined. *N_D. mel_*_WT_ = 10, *N_D. mel_*._KO_ = 10, *N*_Replacement_ = 10, *N_D. sim_*_WT_ = 13, *N_D. sim_*_KO_ = 13. (*C,D*) Relative sperm competitive ability of WT, miR-983 KO, and the replacement strains of *D. melanogaster*. Males of the three genotypes were used as test males. The proportions of sired offspring by test males when WT females were mated first with the test males and subsequently with the reference males were calculated for the defense assay (*C*) and *vice versa* for the offense assay (*D*), respectively. *N_D. mel_*_WT_ = 20, *N_D. mel_*_KO_ = 24 and *N*_replacement_ = 24 for the defense assay; *N_D. mel_*_WT_ = 35, *N_D. mel_*_KO_ = 39 and *N*_replacement_ = 41 for the offense assay. Wilcoxon rank-sum tests were performed and the levels of significance are shown as n.s. for *P* > 0.05, * for *P* < 0.05, and **** for *P* < 0.0001.

Sexual selection often drives rapid evolution of reproductive traits. To understand the impact of miR-983 effects at the phenotypic level, we measured sperm length for each genotype as intensive post-copulatory sexual selection tends to favor exaggerated sperm morphology (Miller and Pitnick 2002; Lüpold et al. 2016). Knocking out miR-983 caused a highly significant decrease in sperm length in *D. melanogaster* (*D. mel* KO vs., *D. mel* WT: 1.724 ± 0.065 mm vs., 1.825 ± 0.047 mm; Wilcoxon rank-sum test, *P* < 2.22 × 10^−16^; fig. 5*A*). The reduction is less severe in the replacement line (1.785 ± 0.079 mm) than in the *D. melanogaster* KO (2.2% vs., 5.5%, Wilcoxon rank-sum test, *P* = 9.7 × 10^−12^; fig. 5*A*). However, no significant difference in sperm length was detected when comparing miR-983 KO with WT in *D. simulans* (*D. sim* KO vs., *D. sim* WT: 1.149 ± 0.033 mm vs., 1.144 ± 0.036 mm, Wilcoxon rank-sum test, *P* > 0.05, fig. 5*A*). MiR-983 KO relative to WT also exhibited increased sperm length variation at the within-individual level in *D. melanogaster* but not in *D. simulans* (fig. 5*B*). We then conducted sperm competition assays for the three genotypes of *D. melanogaster*. MiR-983 KO had a lower defense score compared with WT in *D. melanogaster* (Wilcoxon rank-sum test, *P* < 0.05; fig. 5*C*), which can be rescued by the *D. simulans* miR-983 copy in the replacement line (fig. 5*C*). No difference in offense score was found among the three lines of *D. melanogaster* (Wilcoxon rank-sum test, all *P* > 0.05; fig. 5*D*). These results suggest that miR-983 affects sperm length and plays a significant role in sperm competition in *D. melanogaster* but probably not in *D. simulans*.

As disruptive or diversifying selection may increase expression divergence and lead to enhancing miR-983 and context effects, we reasoned that at each spermatogenetic stage, genes with both enhancing miR-983 and context effects and extremely high (top 5%) expression divergence (*P* < 0.05 based on 10,000 bootstrapping) in the WT are the most likely targets of selection that account for the differential phenotypic effects of miR-983 between *D. melanogaster* and *D. simulans*. We identified 17 such candidates (supplementary table S4, Supplementary Material online), of which eight genes have well-known molecular functions including *globin 1* (*glob1*) involved in the regulation of the cellular level of reactive oxygen species (ROS) and the maintenance of cytoskeletal integrity (Yadav et al. 2015; Yadav and Sarkar 2016), *lectin-46Cb* and *Seminal fluid protein 33A3* (*Sfp33A3*) encoding seminal fluid proteins (SFPs) (Mueller et al. 2004; Findlay et al. 2008), *Troponin C at 47D* (*TpnC47D*) encoding a calcium binding protein subunit (Herranz et al. 2004), *stem cell tumor* (*stet*) encoding an intramembrane protease that functions in the activation of EGFR ligands (Schulz et al. 2002), *Shaw-like* (*Shawl*) encoding part of voltage-gated potassium channel complex (Hodge et al. 2005), *alpha-Esterase-5* (*alpha-Est5*) encoding carboxylesterase (Campbell et al. 2003), and *Coiled-coil domain-containing protein 85B* (*Ccdc85*) functioning as a linker protein in a *serine/threonine phosphatases 1* (*PP1*)-interacting network (Bertran et al. 2019). Particularly, the two SFPs encoding genes, *lectin-46Cb* and *Sfp33A3*, have high expression in the *D. melanogaster* WT but are barely expressed in the *D. simulans* WT or the *D. melanogaster* miR-983 KO at the SG stage (supplementary table S4, Supplementary Material online), probably responsible for the compromised sperm defense ability in the KO line of *D. melanogaster*. In addition, *alpha-Est5* that has a similar pattern of expression divergence at the rST stage was reported to have undergone adaptive sequence evolution in *D. buzzatii* (Piccinali et al. 2007).

## Discussion

Our knowledge about how variants of a single *trans*-regulatory element contribute to long-term divergence is largely limited. One reason for that is antagonistic pleiotropy inherent to *trans* regulators typically hinders adaptation (Orr 2000; Chen and Zhang 2020). Here, we disentangle the regulatory effects of an adaptively evolving and pleiotropic miRNA in *Drosophila* separately from the influence of the genetic background. Moreover, we examine regulatory mode changes that underlie gene expression divergence of *Drosophila* species during spermatogenesis at a single-cyst resolution. To our knowledge, this is the first study that reveals the contribution of miRNA to expression divergence in the framework of *cis* and *trans* regulation. Our results support the view that *trans*-regulatory elements can respond to selection and contribute to divergence of species despite antagonistic pleiotropy (Wagner and Lynch 2008). This study also demonstrates that CRISPR gene editing and single cell RNA-seq are powerful tools to dissect gene-specific effects of *trans* regulators for a comprehensive understanding of gene expression evolution.

Our results show that miR-983 effects under disruptive or diversifying selection promote interspecific gene expression divergence between *D. melanogaster* and *D. simulans* (figs. 2 and 4), echoing the adaptive sequence evolution of miR-983 (Lyu et al. 2014; Mohammed et al. 2014). Nevertheless, we find 3.52 to 17 times more genes affected by miR-983 and the genomic context in the opposite than the same direction during spermatogenesis (fig. 3*C*), consistent with the view that stabilizing selection is the dominant mode of regulatory evolution (Denver et al. 2005; Rifkin et al. 2005; Gilad et al. 2006; Bedford and Hartl 2009; Coolon et al. 2014; Huang et al. 2016; Signor and Nuzhdin 2018). Prior work has shown that stabilizing selection typically constrains variation in gene expression on a genomic scale (Denver et al. 2005; Rifkin et al. 2005; Bedford and Hartl 2009; Coolon et al. 2014; Huang et al. 2016). With the prevalence of stabilizing selection, effects of new *trans* mutations might have been largely absorbed at the gene expression level before they can lead to pleiotropy in protein function. Therefore, both directional and stabilizing selection provide evolutionary resolution for antagonistic pleiotropy at the expression level.

The efficacy of disruptive or diversifying selection is enhanced by stage-specific regulation, as elevated expression divergence for genes with enhancing miR-983 and context effects can only be detected using the single-cyst RNA-seq data, but not the whole-testis data (supplementary fig. S3, Supplementary Material online). Indeed, genes gaining the S mode at individual spermatogenetic stages are enriched in functionalities crucial for each particular stage. Such regulation specificity may not only reduce the number of genes affected at a particular developmental stage, leading to minimized antagonistic pleiotropy but also concentrate on genes involved in coherent functionality, allowing for a better response to natural selection. It was reported that antagonistic pleiotropy is often resolvable in yeast strains that are well adapted to certain environmental factors (Qian et al. 2012). In this sense, miR-983 might have adapted to *in vivo* environments of specific spermatogenetic cell types if considering transcriptomic contexts as the internal environments. The specific regulation of miR-983 during development thus suggests *trans* influences, under sufficient selection, coevolve with *cis* effects to ensure proper regulation programs. Another evidence for coevolution is the higher miR-983 activity in the conspecific than in interspecific context (fig. 2*B,C*). *Trans*-and-*cis* coevolution was also reported for miR-310 s, a cluster of adaptively evolving miRNAs in *Drosophila* (Tang et al. 2010), and many *trans* regulatory proteins (Levine et al. 2007; Rodriguez et al. 2007; Chen et al. 2012; Dai et al. 2021).

Interestingly, regulation specificity in the case of miR-983 is not determined by the *trans* regulator *per se* but likely dependent on the dynamics of transcriptomic contexts. This is like the ubiquitously expressed transcription factors (e.g., Sp1) that gain regulation specificity via cooperation with other cell-specific proteins (Gilmour et al. 2014; O'Connor et al. 2016). At the rST stage, weak miR-983 effects are distributed on a large number of genes gaining S mode, in contrast to the strong miR-983 effects at the SG stage when the vast majority of genes are under conserved regulation. Although it is possible that the overall strength of miR-983 effects is diluted with the increased number of genes it affected, we detected no such correlation across spermatogenetic stages. Alternatively, it is tempting to suggest that miR-983 canalizes genetic programs during spermatogenesis. MiRNAs are thought to act as key players in canalizing development (Hornstein and Shomron 2006), and function both in gene expression tuning and in expression buffering (Wu et al. 2009). The weak miR-983 effects distributed on many genes at rST are concordant with the view that weak regulation of many targets by miRNAs is cumulatively powerful in stability control (Zhao et al. 2017; Zhao et al. 2018; Chen et al. 2019). In support of the suggestion, samples at rST show little variation in expression divergence compared with other stages (fig. 3*B*), albeit the transcriptome at this stage is programed for drastic changes (fig. 4*E*). However, other factors, such as rST being formed within very limited time intervals at the completion of meiosis, may also potentially contribute to the small variation. The validity of the above suggestion needs to be investigated further.

The interactions between miR-983 and context effects provide important insights into the function and evolutionary fates of new *trans* regulators. As *trans* effects are typically subordinate to and antagonize *cis* regulation, the interference of *cis* effects, or context effects in general, should be taken into consideration when exploring functions of *trans* regulators. This is particularly important for young *trans* regulators as new genes are often found “out of the testis” in *Drosophila* and vertebrates (Vinckenbosch et al. 2006; Kaessmann 2010; Chen et al. 2012; Lyu et al. 2014; Zhang and Zhou 2019) or similarly in pollen of rice and *Arabidopsis* (Cui et al. 2015). In the course of spermatogenesis, germ cells experience dynamic chromatin remodeling that leads to more promiscuous transcription (Soumillon et al. 2013; Ernst et al. 2019; Trovero et al. 2020). Given the prevalence of genomic context effects accompanied by rapid transcriptome landscape shifts, the evolutionary fates of nascent *trans* regulators may thus depend not only on their own functions but also on how well they co-evolve with the transcriptome background, as predicted by the Red Queen hypothesis (Van Valen 1973). Thus, the coevolution between miR-983 and the transcriptomic background may support such hypothesis on *de novo* new genes (McLysaght and Hurst 2016; Lu et al. 2018; Zhao et al. 2021). It was suggested that integration of new genes into the extant regulatory networks is a gradual process starting on the network periphery (Zhang et al. 2015). Further studies on the structures of gene regulatory networks during spermatogenesis may shed light on how new *trans* regulators coevolved with genomic context, leading to differential expression profiles between *D. melanogaster* and *D. simulans* in testis.

Finally, our results show that the evolution of a single *trans* element not only has profound impact on gene expression but also leads to phenotypic changes affecting male fitness. The observation that knocking out miR-983 caused reduction in sperm length in *D. melanogaster* but not in *D. simulans* is probably because sperm size in *D. melanogaster* is not canalized as rigidly as in *D. simulans*. A previous analysis reported that *D. simulans* exhibits an extremely stable mode of sperm length distributions across populations while it is more variable in *D. melanogaster* (Joly et al. 2004), suggesting sperm length in *D. simulans* is more robust against environmental and genetic perturbations than in *D. melanogaster*. We found that regulatory mode of miR-983 appears less variable during spermatogenesis in *D. simulans* than *D. melanogaster* (fig. 4*D,E*), which also suggests the developmental process of spermatogenesis is less canalized in the latter species than the former. It is thus not strange that deleting miR-983 has little impact on sperm length in *D. simulans* despite greater expression changes. While what mechanisms account for such difference in the degree of canalization remain unclear, the increased sperm length variation in miR-983 KO of *D. melanogaster* indicate that miR-983 plays a role in canalizing sperm length. The association between sperm length and sperm competition has been extensively investigated (Pitnick et al. 2009; Kahrl et al. 2021). Some evidence suggests that longer sperm is selectively favored in the female sperm storage process in *Drosophila* during sperm-female coevolution (Bressac and Hauschteck-Jungen 1996; Miller and Pitnick 2002). The shorter sperms and decreased sperm defense score of miR-983 KO in *D. melanogaster* suggest that miR-983 has positive contribution to male fitness. However, previous studies found that miR-983 KO mutants have slightly longer sperms than WT in *D. melanogaster* and higher sperm offense score but no difference in defense score in both *D. melanogaster* and *D. simulans* (Lu et al. 2018; Zhao et al. 2021). Such opposing effects may be explained by both the lack of canalization and antagonistic pleiotropy. Consistent with the dual function hypothesis of miRNAs (Wu et al. 2009), genes with enhancing miR-983 and context effects, such as *lectin-46Cb* and *Sfp33A3*, have experienced elevated expression divergence under disruptive or diversifying selection, probably contributing to differential sperm competition abilities. The candidate genes under selection that are identified in this study may thus provide a valuable resource to understand molecular mechanisms underlying sperm length evolution and reproductive isolation in *Drosophila*.

## Materials and Methods

### Construction of MiR-983 Mutant Strains

All flies were raised using the cornmeal-sucrose-yeast medium at 25 °C with a 12-h light-dark cycle throughout this study. A CRISPR/Cas9 system (Cong et al. 2013) was used to generate the miR-983 KO strain of *D. melanogaster* by deleting a 275-bp fragment nearly spanning the entire region of *dme-mir-983-1* and *dme-mir-983-2* from the *w1118* strain. We also replaced the two endogenous *mir-983* copies in *D. melanogaster* with one orthologous copy from *D. simulans*. 15 ug of Cas9 mRNA, 7.5 ug sgRNA, and 15 ug donor DNA were mixed and used for embryo injection. sgRNA targets were designed with CRISPR Optimal Target Finder (http://tools.flycrispr.molbio.wisc.edu/targetFinder/) (Gratz et al. 2014) and amplified by PCR from genomic DNA of the *w1118* strain. We used the MLM3613 (Addgene plasmid 42251) plasmid for cas9 mRNA preparation. To generate the donor pBS-mir983LA-loxp-3xP3-RFP-loxp-mir983RA for the *mir-983* KO, pBluescirpt SK(-) vector was used as the backbone and amplified using the pBF and pBR primers for assembly of homologous arms and the insert. To generate donor pBS-mir983LA-Dsim seq-loxp-3xP3-RFP-loxp-mir983RA for *mir-983* replacement, the precursor sequence of *dsi-mir-983a* from *D. simulans* was inserted into the vector pBS-mir983LA-loxp-3xP3-RFP-loxp-mir983RA between mir983LA and loxp. To remove RFP marker between two loxP sites, flies carrying RFP were crossed with yw, cre; D*/TM3, Sb. Then F1 flies without RFP were balanced with *FM7a*. All the procedures were performed by Fungene Biotech (http://www.fungene.tech/) and detailed in Supplementary Methods.

The miR-983 KO and control stains of *D. simulans* were constructed as described by Zhao et al. (2021). All these five strains were confirmed through DNA sequence by Sanger sequencing and mature miR-983 expression by qRT-PCR. Sequences of the primers used are listed in supplementary table S5, Supplementary Material online.

### MiRNA qRT-PCR

We measured miR-983 expression by miRNA qRT-PCR. Reverse transcription (RT) was performed using TaqMan MicroRNA Assays (Applied Biosystems, USA). qPCR was performed using the Hieff qPCR SYBR Green Master Mix (Yeasen, China). Three biological replicates per genotype and three technical replicates per sample were carried out. Crossing point (C_q_) values were calculated automatically by the Bio-Rad CFX384 Real-Time System. C_q_ values were normalized to the expression level of 2S rRNA which was diluted 1000-fold. Relative expression levels were calculated using the 2^−ΔΔCT^ method (Livak and Schmittgen 2001). Expression differences of miR-983 between genotypes were tested with two-tailed Student's *t*-test. Primers used for qRT-PCR are listed in supplementary table S5, Supplementary Material online.

### RNA-Seq of Adult Testes

Adult flies were allowed to mate three to five days after eclosion and testes were dissected in drops of 1× phosphate-buffered saline (PBS) with two pairs of Dumont #5 forceps. For the extraction of total RNA, 20-25 pairs of testes were pooled as a sample and three biological replicates were collected for each genotype. RNeasy Micro Kit (QIAGEN, German) was used for RNA extraction following the manufacturer's instructions. RNA-seq libraries were constructed and sequenced on a HiSeq X Ten platform (Illumina, USA) generating 150-bp paired-end reads by GENEWIZ (Suzhou, China).

### RNA-Seq for Single Spermatogenic Cysts

Testes from mated male flies two to three days after eclosion were dissected for RNA-seq of single spermatogenic cysts as described above. Two tungsten micro-needles (World Precision Instruments, USA) were used to tear the muscle sheath layer, resulting in germ cell cysts spilling into a drop of 1×PBS. The cysts were viewed using an inverted microscope (Leica DMI4000 microscope) and were picked using a glass capillary (BJ-40, Beijing Zhengtian Yi Science and Trade Co. LTD) pulled by a Sutter instrument P-30 with the following parameters: heat#1 = 930; pull = 909. The cysts were staged using the following criteria: SG, 16-cell cysts with cells of <10 μm in diameter for *D. melanogaster* and < 8 μm in diameter for *D. simulans;* late pSC, 16-cell cysts with cells of 22–25 μm in diameter; rST, cysts with more than 32 round or nearly round cells of 14–16 μm in diameter; and eST, bundles of 250–350 μm in length. Staged single cysts were transferred into PCR tubes by pushing the syringe connected to the glass capillary by an infusion tube. Four to six single cysts were collected, each as a biological replicate, for each developmental stage. Cell lysis, RT, PCR preamplification, and purification of PCR products were conducted using the Discover-sc WTA Kit V2 (Vazyme Biotech, China) according to the user's guides. RNA-seq library was constructed for each cyst using TruePrep DNA Library Prep Kit V2 for Illumina (Vazyme Biotech, China) and an input of 1 ng amplified cDNAs. Sequencing was conducted on a HiSeq X Ten platform (Illumina) by GENEWIZ (Suzhou, China), generating 150-bp paired-end reads.

### RNA-Seq Data Analysis

After removing adapters using Trimmomatic (Bolger et al. 2014) (version 0.36, using parameters 2 : 30 : 10 : 1:true MINLEN:75), short reads were mapped to the corresponding reference genomes of *D. melanogaster* (r6.36) and *D. simulans* (r2.02) using HISAT (Pertea et al. 2016) (version 2.2.0, –dta and other default parameters). Expression abundance of each gene was calculated as Fragments Per Kilobase per Million mapped reads (FPKM) using StringTie (Pertea et al. 2016) (version 2.1.4, used -e and other default parameters). The annotation files of dmel-all-r6.36.gtf and dsim-all-r2.02.gtf, and the list of one-to-one orthologous genes were downloaded from FlyBase (http://flybase.org/) in October 2020. HTSeq (Anders et al. 2015) (version 0.6.0, using parameters -f bam -r name -s no -t exon -m union) was used to count mapped reads for each gene, which were then used as input for intraspecific differential expression analysis using DESeq2 (Love et al. 2014) (version 1.30.0). Pairwise Spearman's correlation coefficient *ρ* of protein-coding gene expression abundance (FPKM) among replicates was calculated to measure the reproducibility of RNA-seq data. We set the cutoff for *ρ* as 0.8. Only a single-cyst sample was removed as an outlier. Only one-to-one orthologous genes with normalized counts (DESeq2) > 5 on average in at least one genotype were considered as expressed and used in further analyses in the focal tissue or spermatogenic stages. The diffusion map of expression abundance was created using the function *DiffusionMap* in the R destiny package (Angerer et al. 2016) (version 3.4.0) based on FPKM values of expressed genes.

### Decomposition of MiR-983 and Genetic Context Effects

We disentangled the regulatory differences among miR-983 variants into miR-983 ortholog and genetic context effects, following the method described by Bao et al. (2019) with modification. Log_2_(fold change) of expression level was calculated with DESeq2 (Love et al. 2014) (version 1.30.0) for each expressed gene in three types of intraspecific comparison: the WT versus miR-983 KO in *D. simulans* (A comparison), replacement versus the *D. melanogaster* KO (B), and the WT versus miR-983 KO in *D. melanogaster* (C). miR-983 ortholog influence was inferred from comparing B and C, while the genetic context effect can be estimated from differences between A and B. The presence of these separate influences was tested using two-tailed Student's *t*-tests based on the log_2_ fold changes of A, B and C, and their standard errors (lfcSE) as calculated by DEseq2. Genes showing the A = B and B ≠ C pattern were classified into the “miR-983 only” category (category I), A ≠ B and B = C genes are “context only” (category II), A ≠ B and B ≠ C genes are “miR-983 & context”, while genes showing the A = B and B = C pattern are called “conserved” (category V). The remaining genes are ambiguous (category VI). Among genes assigned into the “miR-983 & context” category, those showing the (A-B) * (B-C) > 0 pattern are affected by enhancing miR-983 and context interactions (“enhancing”, category III), while those with (A-B) * (B-C) < 0 are under compensating interactions (“compensating”, category IV). To capture any regulatory differences mediated by miR-983, we set no criteria for the degree of difference when comparing A versus B or B versus C but only requiring *P* < 0.05 in Student's *t*-tests. Pearson correlation between A-B and B-C was calculated. As B was used twice when estimating both A-B and B-C, the cross-replicate approach (Fraser 2019) was used to eliminate the potential bias that may cause artifactual negative correlation between them.

### Measurement of Interspecific Expression Divergence

Expression levels (FPKM) of wildtype or miR-983 KO *D. melanogaster* and *D. simulans* strains were used to estimate global expression divergence between species using 1 – *ρ* for testis or each spermatogenic stage, where *ρ* is the Spearman's correlation coefficient between samples from different species. We also performed interspecific differential expression analyses separately for testes or each spermatogenic stage with EBSeq (Leng et al. 2013) (version 1.30.0) which using FPKM values as input.

### MiR-983 Target Prediction

3′UTR sequences of *D. melanogaster* (dmel-all-three_prime_UTR-r6.36.fa) and *D. simulans* (dsim-all-three_prime_UTR-r2.02.fa) were retrieved from FlyBase (http://flybase.org/). Target transcripts were predicted for dme-miR-983-5p and dsi-miR-983a-5p separately using TargetScan (TargetScanFly 7.2) (Agarwal et al. 2018) and the conspecific UTRs. Targets with “7mer-1a,” “7mer-m8,” or “8mer-1a” target site types were retained, and smaller set containing “7mer-m8” or “8mer-1a” types are deemed as stronger targets. Genes encoding targeted transcripts were considered as potential target genes. Predicted miR-983 target genes that are not overlapped between *D. melanogaster* and *D. simulans* were used in this study.

### Clustering of Gene Expression during Spermatogenesis

We clustered genes based on their expression pattern along spermatogenetic stages in the wildtype *D. melanogaster* and *D. simulans* using the Mfuzz R package (Kumar and Futschik 2007) (version 2.50.0, min.std = 0 for the function *filter.std*, m = 1.25 for the function *mfuzz*, min.acore = 0.7 for the function *acore*). The mean FPKM of each gene averaged over replicates from each stage were used as the input. Only one-to-one orthologous genes expressed in at least one stage in *D. melanogaster* or *D. simulans* were used.

### Gene Ontology Analysis

R package GOstats (Falcon and Gentleman 2007) (version 2.56.0) was used for gene ontology enrichment analysis based on the annotation package org.Dm.eg.db (version 3.12.0) for *D. melanogaster*. The 11,299 orthologous genes expressed in testes were used as the background set. The top ten most significantly enriched terms are shown.

### Measuring Sperm Length

Males were separated from females for 4 days until they were used for dissection. One seminal vesicle from each individual was dissected in drops of 1× PBS with two pairs of Dumont #5 forceps. The seminal vesicles were ruptured by gently tightening the forceps. Spilled spermatozoa out of the ruptured seminal vesicle were dispersed as sparsely as possible in a fresh droplet of 1× PBS on a glass slide and covered with a coverslip to take images under a Leica DMI4000 microscope. The lengths of 8–12 sperms per individual and 10 to 13 individuals per genotype were measured in triplicate from images using software LAS V3.8. Coefficient of variation (CV) of sperm length was calculated for each individual as standard deviation divided by the mean (× 100%).

### Measuring Sperm Competitive Ability

Sperm competition assays were conducted for each of the three test *D. melanogaster* lines (miR-983 KO, replacement and WT), respectively, using a similar design as described previously (Amitin and Pitnick 2007). Each virgin *w1118* (*D. mel* WT) female was mated first with a reference red-eyed male (*w1118/Y; miniwhite-UASeGFP/miniwhite-UASeGFP*) and subsequently with a white-eyed test male for the offense assay (test male as P2) and *vice versa* for the defense assay (test male as P1). For the first mating, 3-day-old virgin females were provided with a 3-day-old male each in a fresh vial with live yeast. Males were removed within 1 h of mating. Two days later, the females were provided with a second 3-day-old male in another fresh vial with live yeast. Males were removed within 1 h of mating again. After nine hours, all the females were transferred to a third fresh vial with live yeast, where they were allowed to oviposit for 24 h. About 10 days later, adult progeny from each female in the third vial were collected and scored for eye colors every 2 days. Progeny with red eyes are sired by reference males, while those with white eyes are sired by test males. The relative defense and offense scores were calculated as the proportion of offspring sired by the test male when it is either P1 or P2. Only females that were successfully inseminated by both males survived the whole experiment and had at least 10 progeny were used in this analysis. This experiment was replicated twice and produced consistent results. We present here the results with a larger sample size, of which progeny of 20–24 females were scored for the defense assay and progeny of 35 to 41 females were scored for the offense assay. The Wilcoxon rank-sum test was used to test differences in relative defense or offense scores among genotypes.

### Identifying Potential Targets of Selection Associated with Differential miR-983 Regulation

For each spermatogenetic stage, we resampled the expressed genes with 10,000 bootstrap replicates and recorded the 0.95 quantiles of log_2_FC(*D. mel* WT/*D. sim* WT) of each bootstrap sample. The bootstrap distribution was then used to determine the cutoff of 0.95 quantile of log_2_FC(*D. mel* WT/*D. sim* WT) at a significance level of 0.05. Genes with enhancing miR-983 and context effects (category III) and extremely high (top 5%) interspecific expression divergence (*P* < 0.05 with 10,000 bootstrapping) were considered as potential targets of selection associated with difference of miR-983 regulation.

### Statistical Analysis

All the statistical analysis was finished in R 4.0.3.

## Supplementary Material

Supplementary data are available at *Molecular Biology and Evolution* online.

## Supplementary Material

msac127_Supplementary_Data

## Data Availability

RNA-seq data and relevant processed data files have been submitted to the Gene Expression Omnibus (accession number GSE190885). All other relevant data supporting the key findings of this study are available from the corresponding author upon request.
