# Supplementary material for: Spatiotemporal Regulation of a Single Adaptively Evolving Trans-Regulatory Element Contributes to Spermatogenetic Expression Divergence in Drosophila
Source: Mol Biol Evol. 2022 Jun 10;39(7):msac127. doi: 10.1093/molbev/msac127 (PMC9254010; doi:10.1093/molbev/msac127)
Supplement: msac127_Supplementary_Data [file msac127_supplementary_data.zip › Supplementary_Material.pdf]

Supplementary Material for: Spatiotemporal Regulation of a Single Adaptively Evolving *Trans*-Regulatory Element Contributes to Spermatogenetic Expression Divergence in *Drosophila*

Yumei Huang[1], Rui Shang[1], Guang-An Lu[1], Weishun Zeng[1], Chenglong Huang[1], Chuangchao Zou[1], Tian Tang[1*]

[1]State Key Laboratory of Biocontrol, School of Life Sciences, Sun Yat-sen University, 510275 Guangzhou, Guangdong Province, China.

*Corresponding author, e-mail: lsstt@mail.sysu.edu.cn.

Supplementary Figures

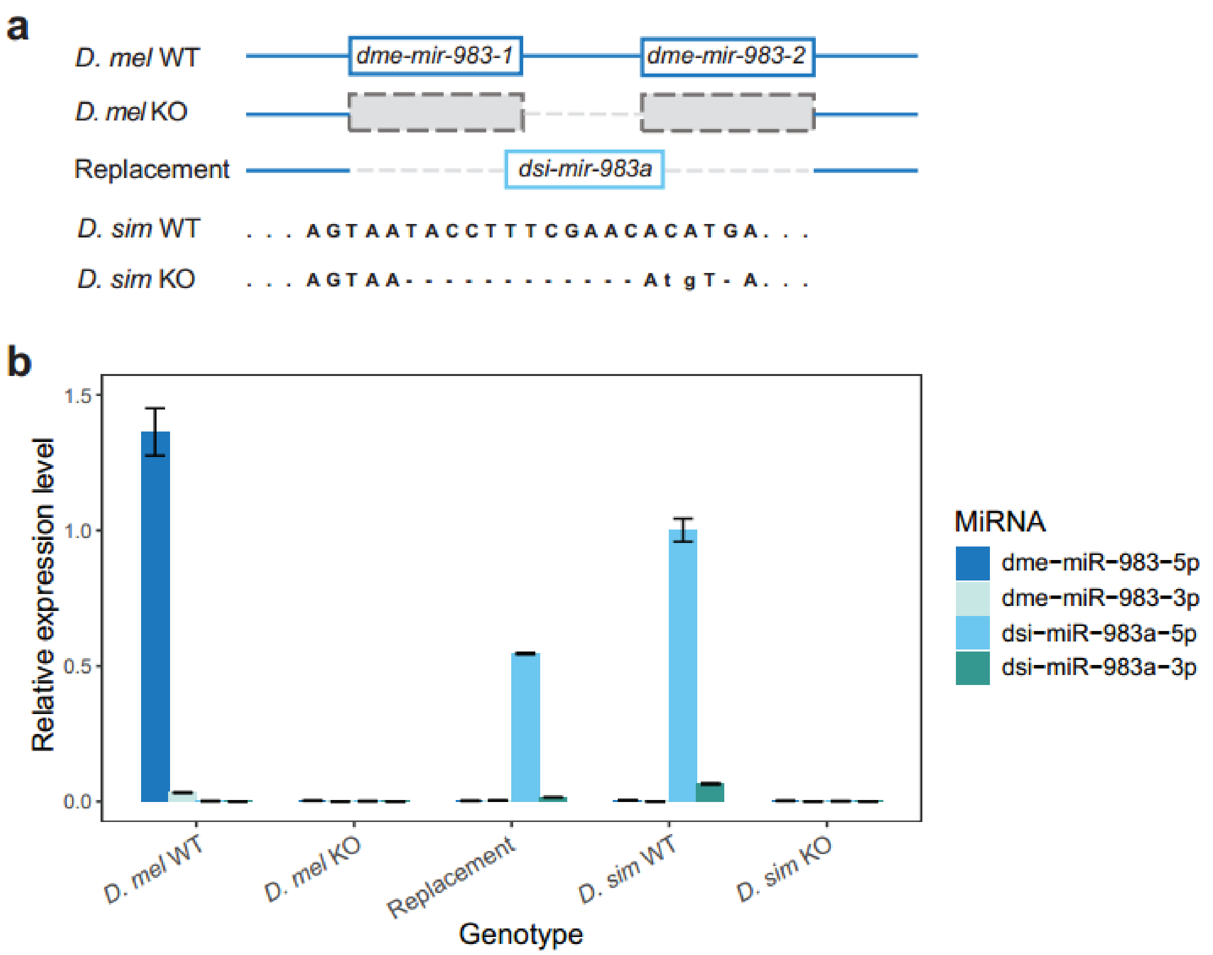

Supplementary Fig. S1. Construction and confirmation of miR-983 fly strains. (*a*) A schematic showing the genetic design for mutant strains. A 275-bp deletion, nearly spanning the two copies of *mir-983*, was generated to construct the *D. mel* KO line. The *dsi-mir-983a* precursor was inserted into the *D. mel* KO background to make the Replacement strain. In the *D. sim* KO strain, an 17-bp region corresponding to dsi-miR-983a-5p accommodates deletion and mutation. (*b*) Relative expression levels of mature miR-983s. The mean expression level of dsi-miR-983a-5p in the strain *D. sim* WT is normalized to 1. Bars represent mean ± SD.

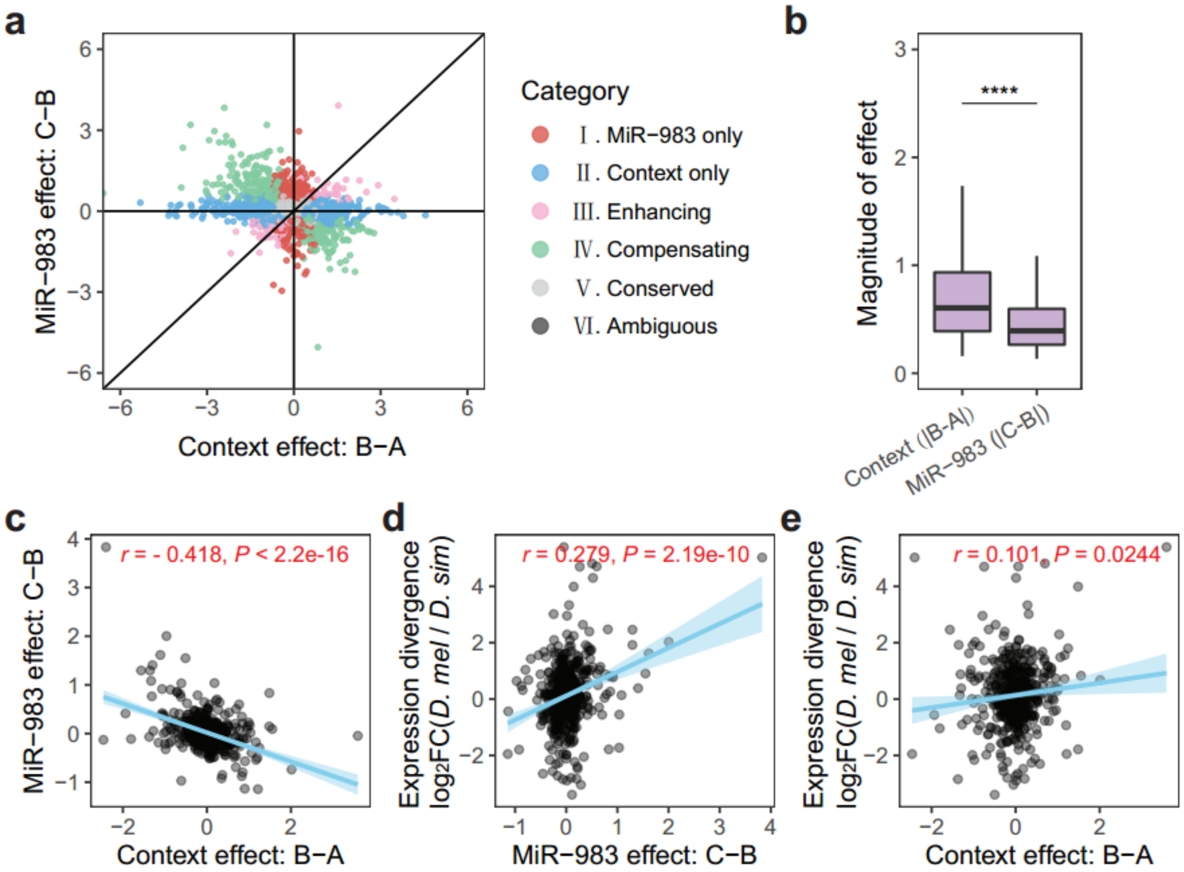

Supplementary Fig. S2. MiR-983 related regulatory effect on testis transcriptome. (*a*) The magnitude of regulatory effects (log_2FC) on each expressed gene. (*b*) Comparison between regulatory magnitudes of context effects on genes under context influence only and miR-983 effects on genes only regulated by miR-983. Wilcoxon rank-sum tests was performed for statistical comparison (****: $P < 0.0001$). (*c*-*e*) Correlation between regulatory components based on targets with “7mer-m8” or “8mer-1a” site types. (*c*) miR-983 effect versus context effect. (*d*) Interspecific expression divergence versus miR-983 effect. (*e*) Interspecific expression divergence versus context effect. Pearson’s correlation coefficients *r* and *P* values are shown.

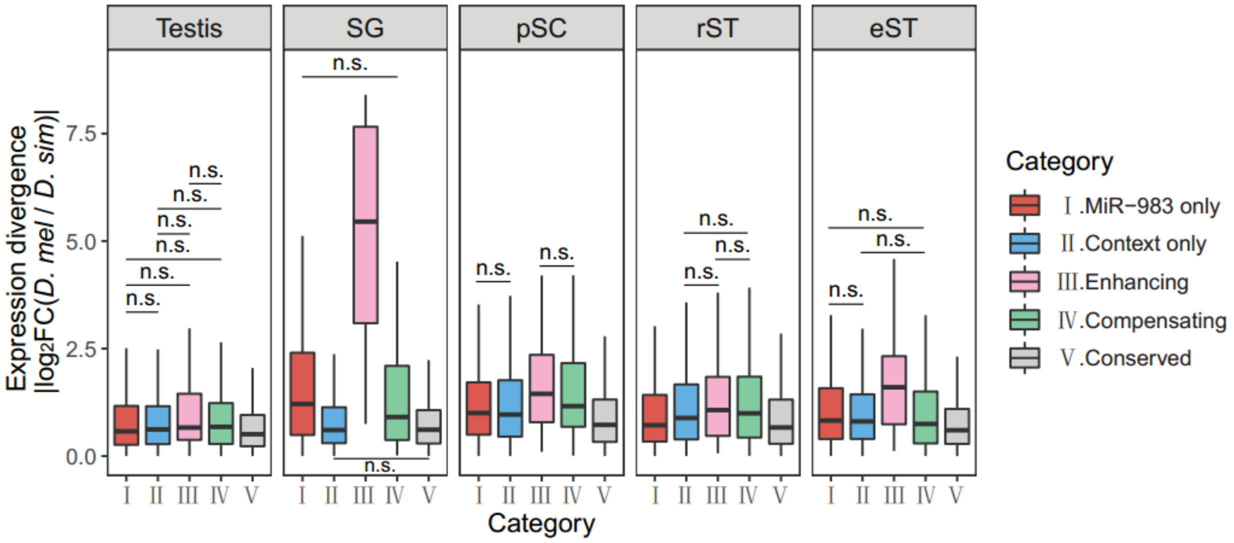

Supplementary Fig. S3. Interspecific expression divergence for genes in different regulatory categories. All pairwise comparisons among categories in the same tissue/stage are significant (Wilcoxon rank-sum test: $P < 0.05$) unless indicated (by n.s.) in the plots.

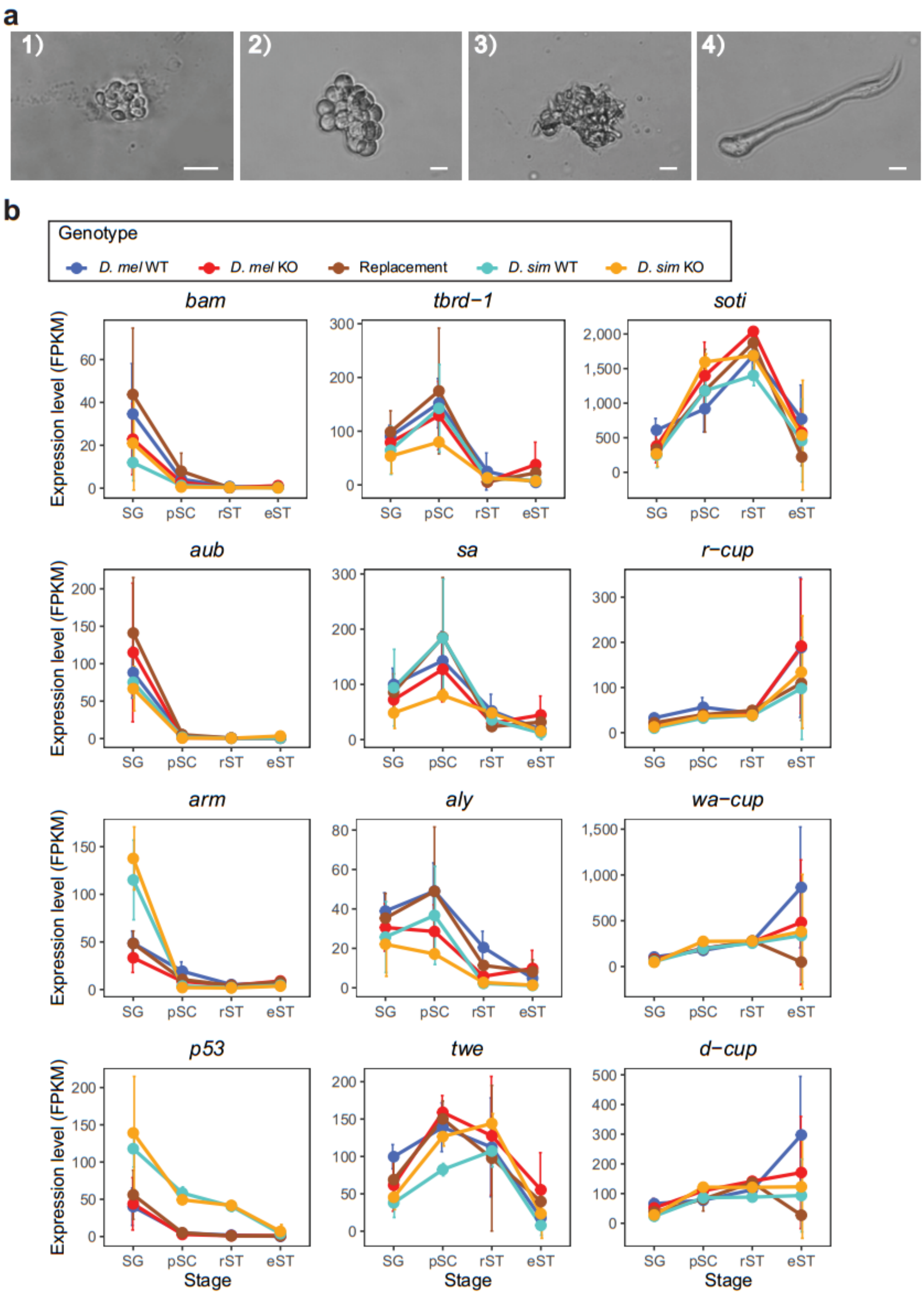

Supplementary Fig. S4. ***Drosophila*** **spermatogenic single cysts and expression patterns of marker genes.** (*a*) Spermatogenic single cysts of *Drosophila melanogaster*. 1) Spermatogonia (SG). 2) Late primary spermatocytes (pSC). 3) Round spermatids (rST). 4) Elongating spermatids (eST).

Scale bars are 20 μm. (*b*) Expression patterns of marker genes during spermatogenesis. The first column presents genes with known specific mitotic expression, the second column contains genes with enriched meiotic expression while the third column represents genes previously reported to be highly expressed at post-meiotic stages. Mean ± SD is shown.

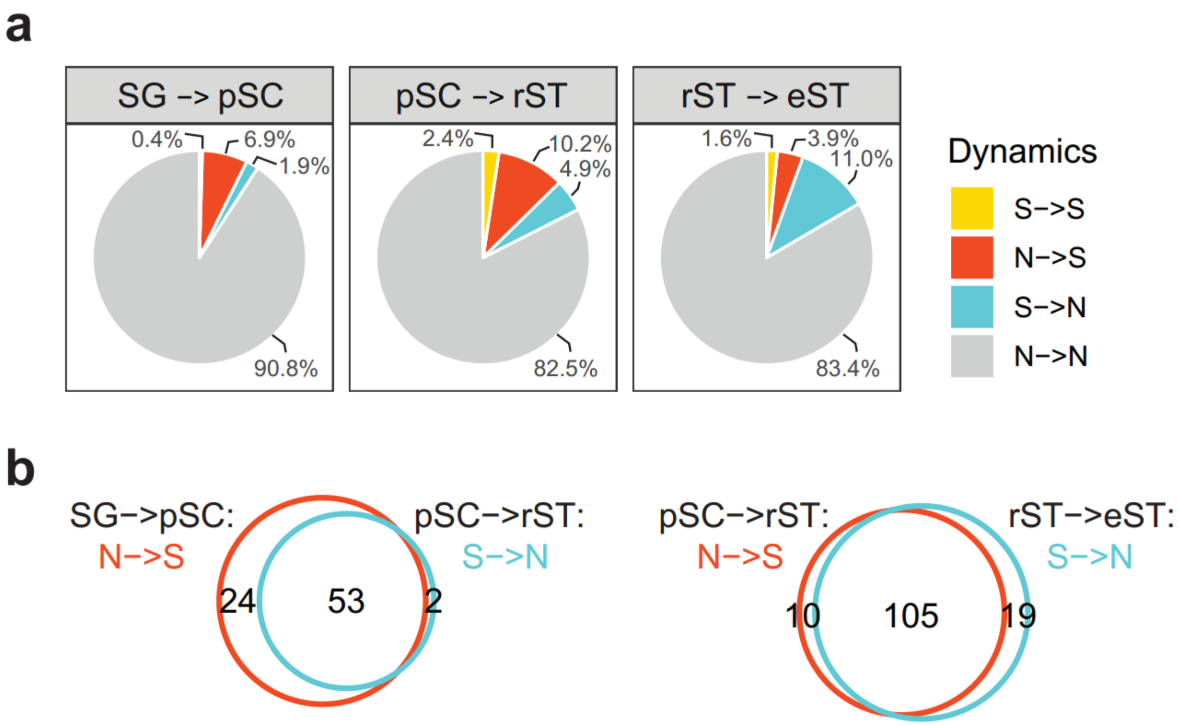

Supplementary Fig. S5. Regulatory dynamics of predicted miR-983 targets across spermatogenesis. (*a*) Pie charts showing proportions of four types of regulatory dynamics between successive stages. “S” comprises “miR-983 only” and “enhancing” categories, while “N” is “context only,” “compensating,” and “conserved.” (*b*) Venn diagrams depicting regulatory mode turnover between successive periods.

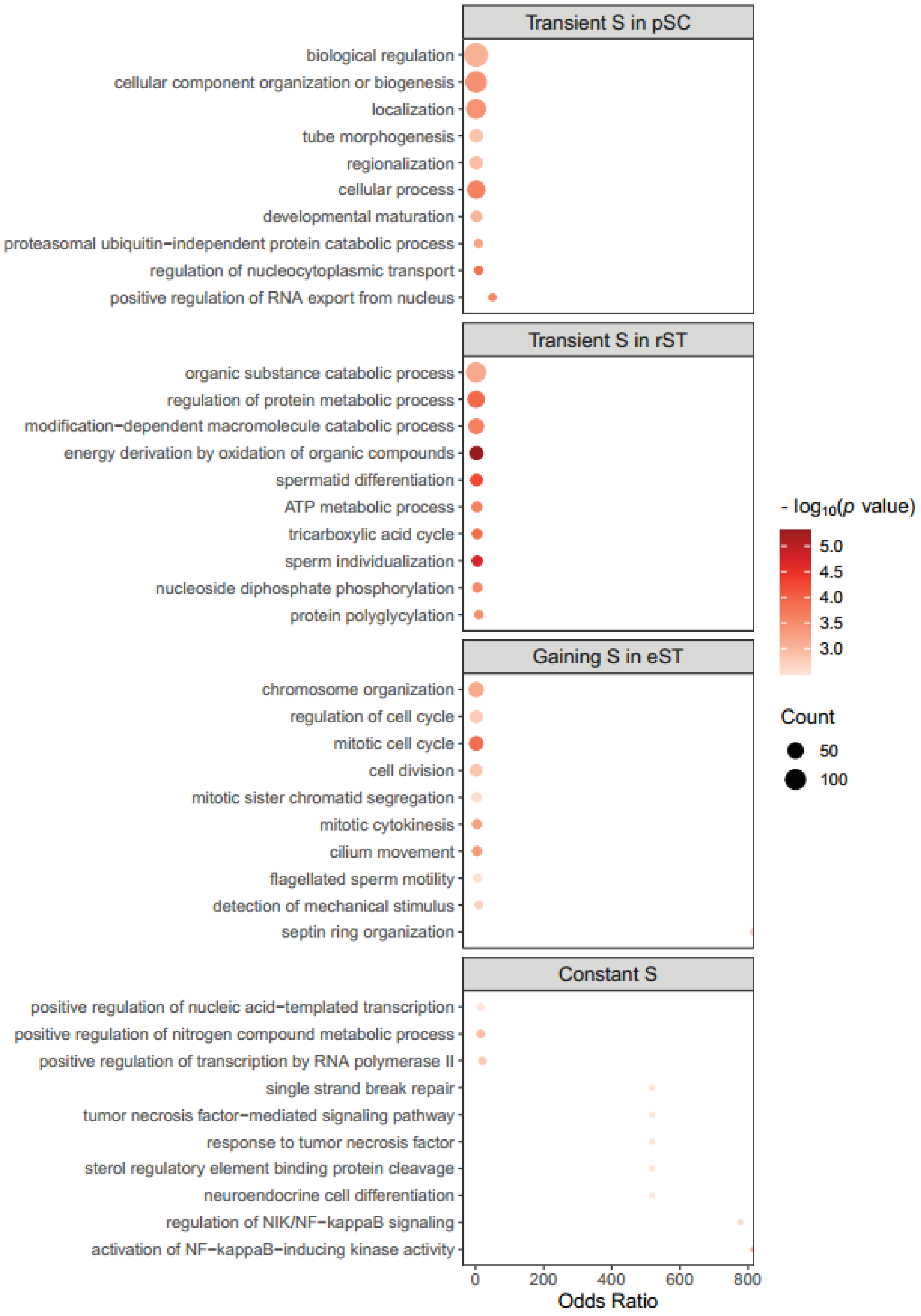

Supplementary Fig. S6. Gene ontology analysis in terms of biological process for genes with particular regulatory dynamics associated with miR-983. "S" denotes regulatory types of "miR-983 only" and "enhancing", while "N" denotes types of "context only," "compensating," and "conserved."

Supplementary Tables

Supplementary Table S1. Summary of RNA-seq data.

[Shown in an independent Excel file]

Supplementary Table S2. Improving estimates of miR-983 effects (C-B) and context effects (B-A) using the cross-replicate approach.

Method	Replicate[a] for (B-A)	Replicate for (C-B)	All unambiguous genes		Targets of miR-983[b]	
			r	*P* value	*r*	*P* value
Standard	1,2,3	1,2,3	-0.314	9.14e-228	-0.418	1.39e-22
Cross-replicate	1	2	-0.512	0	-0.565	2.01e-43
Cross-replicate	1	3	-0.495	0	-0.545	6.38e-40
Cross-replicate	2	1	-0.530	0	-0.612	1.13e-52
Cross-replicate	2	3	-0.507	0	-0.560	1.39e-42
Cross-replicate	3	1	-0.524	0	-0.580	3.31e-46
Cross-replicate	3	2	-0.515	0	-0.542	2.00e-39

[a] Indicate the applied replicates of the "Replacement" genotype.

[b] Non-overlapping targets with “7mer-m8” or “8mer-1a” sites of dme-miR-983 and dsi-miR-983.

Supplementary Table S3. The information of the 10 genes constantly with S mode throughout spermatogenesis.

[Shown in an independent Excel file]

Supplementary Table S4. Genes with enhancing miR-983 and context effects and extremely high expression divergence between *D. melanogaster* and *D. simulans*.

[Shown in an independent Excel file]

Supplementary Table S5. Primer sequences.

[Shown in an independent Excel file]

Supplementary Methods

Construction of the MiR-983 Related Strains of *Drosophila melanogaster*

All the following procedures were performed by Fungene Biotech (http://www.fungene.tech/).

Fly Stocks

All flies used were cultured at 25°C. Genotypes of these flies are:

For injection: *w1118*

For balancing: *FM7a*

3xP3 RFP marker was removed by crossing to Cre-expressing line *yw,Cre; D/TM3,Sb* (BSC#851).

Preparation of sgRNA

sgRNA targets were designed with CRISPR Optimal Target Finder (http://tools.flycrispr.molbio.wisc.edu/targetFinder/) (Gratz, et al. 2014). Genomic DNA was isolated from the injection stock. PCR was performed using primers flanking the targets. And the amplified products were sent for Sanger sequencing. If SNPs were found on the targets, sgRNA sequence would be modified to be consistent with the target sequence of the stock.

Following the protocol of Bassett AR *et al.* (Bassett, et al. 2013), template for *in vitro* transcription by T7 polymerase was generated by annealing two DNA oligonucleotides and PCR amplification. In vitro transcription was performed with the T7 RiboMAX^{TM} Kit (Promega, P1320). Transcripts were purified by phenol-chloroform extraction and isopropanol precipitation.

For *mir-983* knockout and replacement, four sgRNAs were designed to target the gene region. The sgRNAs' sequences were listed below:

Mir-983KI sg1	GTTGTAAACATTCAACTCGA TGG
Mir-983KI sg2	GACACAGTTTATTACAAGAA TGG
Mir-983KI sg3	GGATTTTCAGTTCATTCATT AGG
Mir-983KI sg4	GTAAACATTCAACTCGATGG CGG

Preparation of cas9 mRNA

Plasmid MLM3613 (Addgene plasmid 42251) was linearized with Pme I (NEB, USA) and purified by ethanol precipitation. Cas9 mRNA was transcribed with mMESSAGE mMACHINE® T7 Transcription Kit (Ambion, USA), polyadenylated with the *E.coli* Poly(A) polymerase Kit (NEB, USA), and purified with the RNeasy Mini Kit (QIAGEN, German).

Construction of the donor vector

To generate the donor pBS-mir983LA-loxp-3xP3-RFP-loxp-mir983RA for the *mir-983* knockout, the pBluescirpt SK(-) vector was used as the backbone and amplified with primers pBF and pBR for assembly of homologous arms and the insert.

Using genomic DNA of the *w1118* strain, the left and right homologous arms were amplified with primers mir983-5arm-F and mir983-3arm-R. The amplified backbone, the left homologous arm, and the right homologous arm were first linked together with Gibson Assembly Kit (cat#E2611L, NEB, USA) as pBS-mir983LA-mir983RA. The loxp-3xP3-RFP-loxp cassette was amplified from pBSK-LRL (from Fungene Biotech, unpublished) with primers LRL-F and LRL-R. mir983LA-pBS-mir983RA linear DNA without the *mir-983* gene region sequence was amplified from pBS-mir983LA-mir983RA vector with primers mir983-insert-F and mir983-insert-R and combined with the loxp-3xP3-RFP-loxp cassette using a Gibson Assembly Kit producing the final construct pBS-mir983LA-loxp-3xP3-RFP-loxp-mir983RA. The *mir-983* gene region sequence was thus replaced by the loxp-3xP3-RFP-loxp cassette.

Primers:

pBF	TGGCGTAATCATGGTCATAGC
pBR	CTGGCGTAATAGCGAAGAGG
mir983-5arm-F	CCTCTTCGCTATTACGCCAGCCGTGTGGGTAATGTTCAGC
mir983-3arm-R	GCTATGACCATGATTACGCCAGCGATGTGGAGTGCGAGTA
LRL-F	ttcgctattacgccagataac
LRL-R	gaccatgattacgccaataac
mir983-insert-F	gttattggcgtaatcatggtcCGCTGGCGTATGAGAAATTACTTA
mir983-insert-R	gttatctggcgtaatagcgaaATAGCATATGCATATAATACGAA

To generate the pBS-mir983LA-Dsim seq-loxp-3xP3-RFP-loxp-mir983RA donor for *mir-983*

replacement, the *dsi-mir-983a* precursor sequence from *D. simulans* was inserted into the vector pBS-mir983LA-loxp-3xP3-RFP-loxp-mir983RA between mir983LA and loxp.

The 120-nt precursor sequences of *dsi-mir-983a* from *D. simulans* are shown below:
TTGCTTTGCATATGATATCTTGCAGTAATTTAGTAATACCTTTCGAACACATGATTTTCAGT
CATTCATGAGTTCGTCAGGTATTATCTAGTTATTGTAAACATTCAACTCGCTGGCGGA

Embryo Injection and Screening

15 ug of Cas9 mRNA, 7.5 ug sgRNA and 15 ug donor DNA were mixed with DEPC water in a 30-uL volume. And the mix was used for embryo injection. Embryos were injected according to standard protocols at 18°C and were shifted to 25°C immediately after injection. When the injected P0 embryos grew into adults, they were crossed with *FM7a*. F1 flies were then screened for candidates that carried RFP in eyes under a fluorescence stereo microscope.

Molecular Characterization of loxp-3xP3-RFP-loxp Knockin

To verify that loxp-3xP3-RFP-loxp was inserted as expected, two pairs of primers were designed outside each homologous arm. Flies with RFP were homogenized for genomic DNA extraction and PCR amplification. If DNA products were in the expected length and confirmed by Sanger sequencing, the correct loxp-3xP3-RFP-loxp-knockin transgenic line was obtained.

Excision of RFP Marker

To remove RFP marker between two loxP sites, flies carrying RFP were crossed with *yw, cre; D*/TM3, Sb*. Then F1 flies without RFP were balanced with *FM7a*.

References

Bassett AR, Tibbit C, Ponting CP, Liu JL. 2013. Highly efficient targeted mutagenesis of *Drosophila* with the CRISPR/Cas9 system. *Cell Rep.* 4(1):220-228.

Gratz SJ, Ukken FP, Rubinstein CD, Thiede G, Donohue LK, Cummings AM, O'Connor-Giles KM. 2014. Highly specific and efficient CRISPR/Cas9-catalyzed homology-directed repair in *Drosophila*. *Genetics* 196(4):961-971.
